# Prevalence of survival without major morbidity and associated risk factors among very preterm infants: a systematic review and meta-analysis

**DOI:** 10.3389/fped.2025.1628472

**Published:** 2025-08-18

**Authors:** Xiangtong Zhang, Peiqin Wang, Zhi Wan, Ping Xiong, Dandan Rao, Zhangbin Yu, Genfeng Wu

**Affiliations:** ^1^ Department of Pediatrics, Longgang District Maternity & Child Healthcare Hospital of Shenzhen City (Longgang Maternity and Child Institute of Shantou University Medical College), Shenzhen, Guangdong, China; ^2^ Department of Neonatology, The Central Hospital of Enshi Tujia and Miao Autonomous Prefecture, Enshi, Hubei, China; ^3^ Department of Neonatology, Shenzhen Luohu People’s Hospital, Shenzhen, Guangdong, China; ^4^ Department of Neonatology, Shenzhen People’s Hospital, The Second Clinical Medical College of Jinan University, First Affiliated Hospital of Southern University of Science and Technology, Shenzhen, Guangdong, China

**Keywords:** very preterm infants, survival, morbidity, prevalence, risk factor, meta-analysis

## Abstract

**Introduction:**

Survival without major morbidity (SWMM) in very preterm infants represents a critical outcome measure in neonatal care. This systematic review evaluates both the prevalence of SWMM among infants born before 32 weeks' gestation and the associated risk factors.

**Methods:**

We conducted a comprehensive search of PubMed, Web of Science, Embase, Cochrane Library, Scopus, CNKI, CBM, and Wanfang databases from inception through February 4, 2025. Two independent reviewers performed study selection and data extraction. Study quality was assessed using the Agency for Healthcare Research and Quality (AHRQ) checklist for cross-sectional studies and the Newcastle-Ottawa Scale (NOS) for cohort studies. Pooled prevalence was calculated using a random-effects model. Heterogeneity was explored through subgroup analyses and meta-regression, and publication bias was assessed via funnel plots and further evaluated with trim-and-fill analysis. Risk factors were evaluated using multivariate meta-analysis of adjusted odds ratios (ORs) with 95% confidence intervals (CIs).

**Results:**

From 1,606 screened articles, 35 studies spanning twelve countries met inclusion criteria. The pooled SWMM incidence was 47% (95% CI: 40%–54%), with notable gestational age stratification: 67% (95% CI: 62%–72%) for infants <32 weeks vs. 44% (95% CI: 26%–61%) for those <28 weeks. Meta-analysis identified gestational age maturity (OR: 1.65; 95% CI: 1.50–1.81), antenatal corticosteroid administration (OR: 1.46; 95% CI: 1.12–1.89), and higher 5-minute Apgar scores (OR: 1.21; 95% CI: 1.06–1.37) as positive predictors of SWMM. Conversely, male sex (OR: 0.62; 95% CI: 0.55–0.71) and hemodynamically significant patent ductus arteriosus (OR: 0.51; 95% CI: 0.38–0.69) showed negative associations with SWMM.

**Conclusion:**

The review reports a 47% SWMM rate among very preterm infants, with higher rates observed in infants of later gestational age. Key predictors include gestational age, 5-minute Apgar score, and antenatal corticosteroids, while male sex and patent ductus arteriosus are associated with reduced SWMM. Limitations include heterogeneity in SWMM definitions and geographic variability. Future research should focus on standardizing outcome measures and validating risk factors through multinational studies.

**Systematic Review Registration:**

https://www.crd.york.ac.uk/PROSPERO/view/CRD42025641924, PROSPERO CRD42025641924.

## 1 Introduction

Prematurity remains the leading cause of neonatal mortality worldwide. With advancements in perinatal and neonatal intensive care, the survival rates of very preterm infants (VPIs, <32 weeks gestation) have improved markedly in recent decades (1–3). However, very preterm infants are characterized by physiological immaturity, making them particularly vulnerable to severe short- and long-term complications, including intraventricular hemorrhage (IVH), necrotizing enterocolitis (NEC), bronchopulmonary dysplasia (BPD), and retinopathy of prematurity (ROP) (4). These conditions not only elevate the risk of mortality during the neonatal period but also impose lifelong consequences on neurodevelopment, cognitive function, and quality of life (5, 6).

The composite outcome “survival without major morbidity” (SWMM) has emerged as an important measure of neonatal care quality, reflecting both mortality avoidance and minimization of severe complications (7–10). Contemporary neonatal research has operationalized SWMM to typically include the absence of mortality along with major morbidities such as BPD, severe IVH (grade ≥3), NEC (stage ≥2), late-onset neonatal infection, and severe ROP (stage ≥3 or requiring treatment) (11). However, significant gaps remain in our understanding of SWMM epidemiology. International comparisons are hindered by variability in diagnostic thresholds, therapeutic approaches, and data collection methods (12–16). Furthermore, the relative contributions of biological and clinical factors to SWMM outcomes remain poorly quantified, with inconsistent reporting of variables such as gestational age, sex differences, and treatment effects across existing studies (17–21).

This study aims to provide a comprehensive analysis of SWMM in very preterm populations through two primary objectives: first, to conduct a meta-analysis estimating the global incidence of SWMM among very preterm infants, including those who are extremely preterm, while examining international variations and temporal trends; second, to identify risk factors associated with SWMM through a critical appraisal of existing evidence. By synthesizing evidence from multinational cohorts, we seek to inform clinical practice and facilitate more standardized outcome assessment in neonatal research.

## 2 Materials and methods

### 2.1 Study design and registration

This review was conducted in accordance with the Preferred Reporting Items for Systematic Reviews and Meta-Analysis (PRISMA) statement guidelines (22). The study was registered with the International Prospective Register of Systematic Reviews (PROSPERO; registration number: CRD42025641924). Approval from an Ethics Committee was not required, as the study is based entirely on previously published research.

### 2.2 Search strategy

A comprehensive search of the literature was conducted across eight electronic databases: PubMed, Embase, the Cochrane Library, Web of Science, Scopus, the China National Knowledge Infrastructure Database (CNKI), the Chinese Biomedical Database (CBM), and Wanfang. The search spanned from the inception of each database to February 4, 2025, utilizing keywords, Medical Subject Headings (MeSH), and other index terms, as well as combinations of these terms and their appropriate synonyms. Additionally, we manually examined the reference lists of the identified articles and used Science Citation Index to do forward citation tracking of included studies. Additional file S1 presents the comprehensive search plan. All retrieved records were imported into an EndNote library. Two investigators independently screened all articles for eligibility. In cases where consensus could not be reached, a third investigator reviewed the full text of the article to resolve any disagreements.

### 2.3 Eligibility criteria

All included studies met the following inclusion criteria: (1) study design: observational studies, including cross-sectional studies and prospective/retrospective cohort studies; (2) study participants: very preterm infants, including those who are extremely preterm, with a gestational age of less than 32 weeks; (3) definition of major morbidity: Studies must provide clear definitions for all of the following conditions: chronic lung disease (CLD)/bronchopulmonary dysplasia (BPD), severe neurological injury (SNI), necrotizing enterocolitis (NEC), retinopathy of prematurity (ROP), with or without sepsis; (4) outcome indicators: studies were required to define SWMM as discharge survival without any of the four specified morbidities (CLD/BPD, SNI, NEC, ROP), with or without sepsis, and to report either calculable SWMM rates (number of SWMM cases/total participants) or analyze risk factors for SWMM or death/major morbidity (DOMM); and (5) sample size: studies must have a sample size greater than 100 participants. Additional file S2 contains the details of the eligibility criteria and relevant definitions.

The composite outcome SWMM reflects critical neonatal care quality and long-term prognosis. To address heterogeneity in published definitions (7, 10, 23, 24), we prioritized studies using two prevalent frameworks: Definition A (CLD/BPD, SNI, NEC, ROP > stage 2) and Definition B (Definition A + sepsis). Studies employing either definition were included, with subgroup analyses to assess definition-dependent variations.

The exclusion criteria were as follows: (1) studies not published in Chinese or English; (2) duplicate publications, abstracts, entries in clinical trial registries, case reports, conference proceedings, reviews, letters, and editorials; (3) studies from which outcome indicator data could not be extracted (no specific number of SWMM events); (4) studies in which outcome indicators were measured after hospital discharge; and (5) studies for which full-text articles were unavailable.

### 2.4 Data extraction

All data were independently extracted from the included studies by two researchers, who subsequently cross-checked the information. The following details were recorded: the first author's name, publication year, year of investigation, study location, sample size of the target population, definition of SWMM or the diagnostic criteria for major morbidity, prevalence of SWMM (specifically including both the number of cases with SWMM and the denominator used in the original studies for calculating the SWMM rate, which may vary as either the number of survivors or the total population including deceased individuals), and risk factors associated with SWMM or DOMM. All extracted data were stored in Microsoft Excel. Any disagreements were resolved through mutual discussion among the authors.

### 2.5 Quality assessment of studies

Two independent reviewers assessed the quality of the included studies using two distinct tools: the Agency for Healthcare Research and Quality (AHRQ) checklist for cross-sectional studies and the Newcastle-Ottawa Scale (NOS) for cohort studies (25, 26). The AHRQ checklist, which comprises 11 items, rates each item as “yes,” “no,” or “unclear.” Higher scores indicate a lower risk of bias, with studies categorized as low quality (score 0–3), moderate quality (score 4–7), or high quality (score 8–11). The NOS, which includes 8 items related to selection, comparability, and outcomes, classifies studies into three quality categories based on their total score: 0–3 for low quality, 4–6 for moderate quality, and 7–9 for high quality. A third reviewer extracted data from five randomly selected studies and evaluated their methodological quality and risk of bias to ensure the accuracy of the assessment. Additional file S3 provides the guidelines for evaluating the quality of the grading method.

### 2.6 Data analysis

The sample size of very preterm infants and the number of cases with SWMM were extracted. The Metaprop package in Stata version 17.0 (Stata Corp.) was used to calculate the pooled prevalence with 95% confidence interval (CI). The pooled prevalene estimates are expressed as percentages, along with 95% prediction intervals (PIs). A *p*-value of less than 0.05 was considered statistically significant. Heterogeneity among studies was assessed using Cochran's Q statistic, and the degree of heterogeneity was quantified with the *I*^2^ statistic, where *I*^2^ values of 25%, 50%, and 75% indicated low, moderate, and high heterogeneity, respectively. Given the inherent heterogeneity of prevalence data, a random-effects model was applied. The findings are presented in the form of forest plots. In an attempt to understand the heterogeneity sources, the subgroup and meta-regression were analyzed. In the stratified meta-analyses, the literature was categorized into subgroups based on various factors, including gestational age ranges, study periods, sample sizes, study regions, diagnoses of major morbidity, and denominators for SWMM rate calculations. To ensure the stability and consistency of our findings, we conducted a sensitivity analysis by excluding one study at a time. Publication bias was evaluated using a funnel plot, and asymmetry was tested with Egger's linear regression method. If publication bias was detected, we adjusted the prevalence rate using the “metatrim” command with the trim-and-fill method. A significant level of 0.05 was set for statistical analysis.

Each reported risk factor was synthesized qualitatively. The total number of low- and moderate-risk bias studies, along with the percentage of studies demonstrating positive correlations, were used to classify them as definite, likely, unclear, or not risk factors (see additional file S4). For risk factors with sufficiently homogeneous definitions and reference ranges, a quantitative meta-analysis of low- and moderate-risk-of-bias studies was conducted to estimate a combined odds ratio (OR). A random-effects meta-analysis was employed *a priori* due to anticipated variations in study population, design, period, sample size, geography, and other factors. As a general rule, we included only risk factors that were investigated in at least three studies using a multivariate design. For studies reporting ORs for DOMM, these effect estimates were converted to reflect the corresponding association with SWMM through reciprocal transformation (i.e., OR_SWMM = 1/OR_DOMM). This transformation facilitated a pooled analysis of all studies using a consistent outcome direction. The converted ORs were subsequently analyzed along with their corresponding standard errors using generic inverse-variance weighting in our random-effects meta-analysis model. Ultimately, seven factors (gestational age, male sex, birth weight, antenatal steroids, cesarean section, 5 minute Apgar score, and patent ductus arteriosus) met our criteria and were included in the meta-analysis.

## 3 Results

### 3.1 Study process

**Figure 1:**
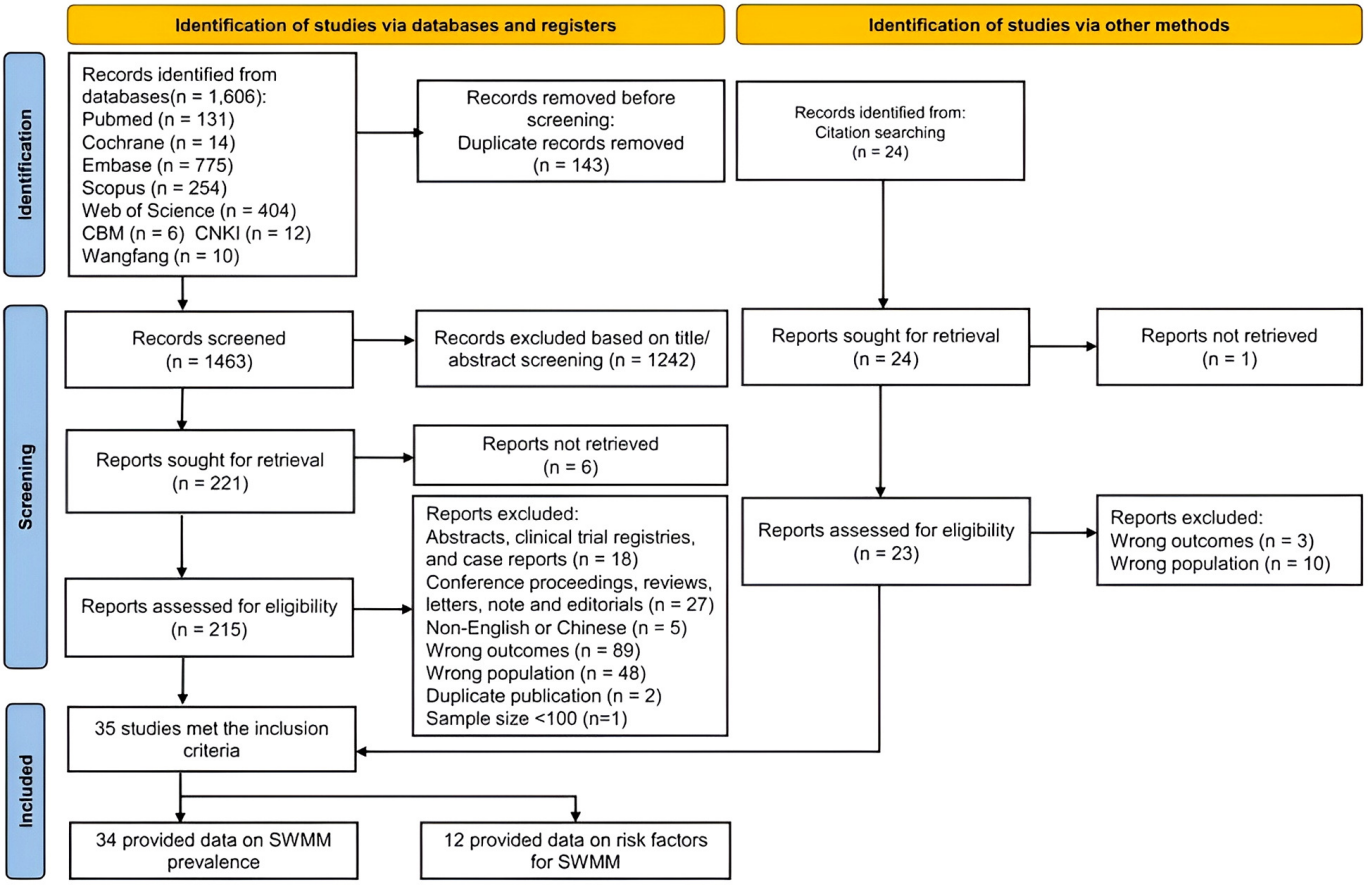
PRIMSA flow diagram.

The literature search yielded 1,606 articles (see Figure 1). After removing duplicates, 1,463 studies remained. Following the screening of titles and abstracts, we identified 221 articles that were eligible for full-text review. Ultimately, a total of 35 studies met the inclusion criteria for the comprehensive review and meta-analysis (3, 12, 13, 19, 25–57). Among these 35 included studies, thirty-four were suitable for the meta-analysis of the prevalence of SWMM, while 12 were eligible for the meta-analysis of risk factors associated with SWMM (19, 27, 28, 30, 38, 40, 41, 44, 47, 48, 53, 56).

### 3.2 Characteristics of the included studies

The characteristics of the 35 studies included in the systematic review are presented in Table 1. The studies originated from various countries, including Austria (40), Canada (30, 35, 54), China (13, 19, 31, 32, 36, 37, 42–44, 52, 53, 56, 57), France (12), Korea (41), New Zealand (32), the United Kingdom (31, 43), the United States (3, 29, 50, 51, 55), Singapore (27, 28), Sweden (38, 39, 49), Switzerland (46), and Türkiye (39). Among these, two studies were cross-sectional (37, 39), while thirty-three were cohort studies (3, 12, 13, 19, 27–36, 38, 40–57). The sample sizes varied significantly, ranging from 196 to 34,636, with the two largest studies comprising 16,679 infants (3) and 34,636 infants (51). Of the 34 studies that reported the proportion of SWMM, which collectively included a total sample size of 202,371 infants, 10 studies used the number of survivors as the denominator for calculating the proportion of SWMM (12, 29, 32, 33, 37–39, 41, 46, 50). In contrast, the remaining 24 studies utilized either the number of infants admitted to the neonatal intensive care unit or the total number of infants included in the study as the denominator (3, 13, 19, 27, 28, 30, 31, 34–36, 40, 42–45, 47–49, 51–57).

**Table 1:** Characteristics of included studies and quality assessment results.

Authors	Year of publication (data)		Region	Study population (GA, weeks)	sample size (GA < 32 weeks)	SWMM rate (%)	Risk factor associated with SWMM or DOMM	study quality score
Agarwal et al. (28)	2015	(2000–2009)	Singapore	<29	835	370 (44.31)	1–14	8
Agarwal et al. (27)	2013	(1990–2007)	Singapore	22–26 6/7	570	138 (24.21)	1, 5–8, 10–13	8
Ancel et al. (12)	2015	(2011)	France	22–34	1010	598 (59.21)	NA	8
Anderson et al. (29)	2016	(2007–2011)	USA	22–28	4,351	1,410 (32.41)	NA	7
Beltempo et al. (30)	2019	(2010–2015)	Canada	22–28	9,240	3,381 (36.59)	15	7
Cao et al. (31)	2021	(2019)	China	<32	8,171	4,677 (57.24)	NA	8
Chang et al. (32)	2018	(2007–2011)	China	22–26	647	81 (12.52)	NA	8
Costeloe, et al. (33)	2012	(2006)	UK	22–26	1,041	423 (40.63)	NA	8
Cust et al. (34)	2003	(1998)	New Zealand	“high risk” %	1,241	898 (72.36)	NA	8
Higgins et al. (3)	2024	(2011–2019)	USA	22–28	29,570	10,054 (34.00)	NA	7
Isayama et al. (35)	2012	(2006–2008)	Canada	BW <1,500 g	13,745	9,095 (66.17)	NA	8
Jiang et al. (37)	2024	(2019–2021)	China	22–25	3305	2003 (60.61)	NA	9
Jiang et al. (36)	2020	(2015–2016)	China	<34	392	45 (11.48)	NA	8
Johanzon et al. (38)	2008	(1998–2003)	Sweden	22–27 6/7	194	83 (42.78)	1- 4, 6, 8, 9, 16–20, 22, 23	8
Kavurt et al. (39)	2023	(2017–2021)	Türkiye	ELBW	263	98 (37.26)	NA	7
Kiechl-Kohlendorfer et al. (40)	2019	(2011–2016)	Austria	23–31 6/7	5,198	4,065 (78.2)	1, 3, 6–8	8
Kim et al. (41)	2019	(2013–2016)	Korea	23–24	1,382	293 (21.20)	1, 2, 4–7, 24, 25	8
Kong et al. (42)	2016	(2013–2014)	China	24–31 6/7	1,760	1,313 (74.60)	NA	8
Li et al. (43)	2024	(2019)	China	24–31 6/7	8,380	4,604 (54.94)	NA	7
Li et al. (44)	2024	(2022)	China	<32	671	450 (67.06)	1–3, 18, 26–30	7
Marlow et al. (45)	2015	(2006)	UK	22–26	2,460	246 (10.00)	NA	7
Morgillo, et al. (46)	2014	(2000–2019)	Switzerland	23–27 6/7	160	121 (75.63)	NA	8
Nourkami-Tutdibi et al. (47)	2021	(2011–2012)	EPICE	22–31 6/7	7,607	5,206 (68.44)	16	7
Pan, et al. (19)	2023	(2019–2021)	China	< 32	2900	2,391 (82.45)	1–8, 12, 14, 16, 18, 26–28, 30–44	8
Peng et al. (13)	2022	(2018–2020)	China	22–31 6/7	807	530 (65.68)	NA	8
Serenius et al. (48)	2004	(1992–1998)	Sweden	23–25	213	75 (35.21)	1–9, 12, 16, 18, 19, 45–48	8
Simic et al. (49)	2014	(2004–2007)	Sweden	<27	321	172 (53.58)	NA	8
Stoll et al. (50)	2010	(2003–2007)	USA	22–28	9,575	3,543 (37.00)	NA	8
Stoll et al. (51)	2015	(1993–2012)	USA	22–28	66,122	16,052 (24.28)	NA	8
Wu et al. (52)	2021	(2018–2019)	China	<32	2,339	1,507 (64.43)	NA	8
Ye (53)	2024	(2019–2021)	China	<32	650	407 (62.62)	2, 7, 47, 49	8
Yeung et al. (54)	2024	(2010–2021)	Canada	23–27 6/7	8,180	2,621 (32.04)	NA	6
Zayek et al. (55)	2011	(1998–2008)	USA	22–26 6/7	790	368 (46.58)	NA	7
Zhu et al. (57)	2021	(2010–2019)	China	<28	8,281	780 (9.42)	3, 6, 7, 36, 50–52	8

1, antenatal corticosteroids; 2, cesarean section; 3, multiple birth; 4, chorioamnionitis; 5, 5 min Apgar score; 6, gestational age; 7, birth weight; 8, gender; 9, small for gestational age; 10, early hypotension needing inotropes; 11, air leaks; 12, persistent ductus arteriosus; 13, respiratory distress syndrome; 14, CRIB-II score, Clinical Risk Index for Babies-revised version II; 15, SNAP-II cut-off = 14; 16, maternal age; 17, previous preterm birth; 18, premature rupture of membranes; 19, *in utero* transfer; 20, tocolysis; 21, antibiotic treatment; 22, breech presentation; 23, time delivery-discharge; 24, body temperature; 25, mortality rate-dependent variations(≤50% vs. ≥50%); 26, antenatal magnesium sulfate; 27, gestational diabetes mellitus; 28, assisted reproduction status; 29, surfactant therapy; 30, DR resuscitation; 31, asphyxia; 32, tachycardia; 33, persistent pulmonary hypertension; 34, atrial septal defect; 35, ventricular septal defect; 36, 1 min Apgar score; 37, SNAP-II, Score for Neonatal Acute Physiology, version II; 38, SNAPPE-II, Score for Neonatal Acute Physiology-II and Perinatal Extension; 39, white blood cells in peripheral blood; 40, gestational hypertension; 41, intrahepatic cholestasis of pregnancy; 42, hypothyroidism during pregnancy; 43, GBS infection; 44, abnormal amniotic fluid; 45, placental abruption; 46, pre-eclampsia; 47, duration of MV; 48, time period 3 (September 1996 to December 1998) vs. time periods 1 and 2 combined (January 1992 to August 1996); 49, duration of central venous catheterization; 50, maternal obesity; 51, antepartum hemorrhage; 52, neonatal blood pH <7.2; %: born at < 32 weeks gestation or < 1,500 g birth weight, or received assisted ventilation for four hours or more, or had major surgery.

### 3.3 Quality of the included studies

The quality assessment of the included studies is presented in additional file S5. The quality scores of the included articles ranged from 6 to 9 points. Two studies were categorized as having a moderate risk of bias, while the remaining 33 studies were classified as having a low risk across all domains and were considered to be at an overall low risk of bias.

### 3.4 Meta-analysis results

#### 3.4.1 Total prevalence

**Figure 2:**
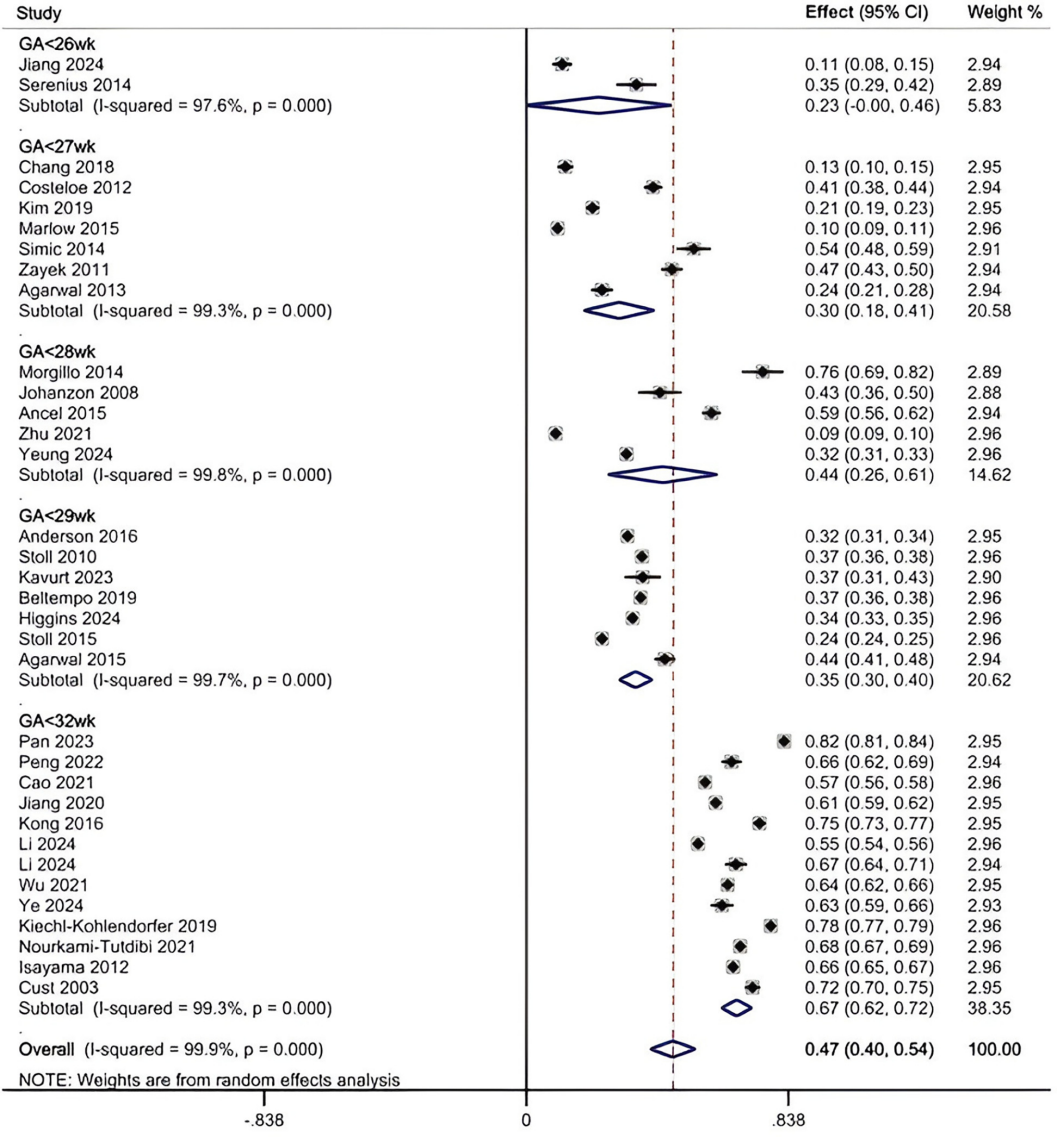
Pooled prevalence of SWMM by random-effects meta-analysis. There were 34 studies for synthesizing the prevalence of SWMM in VPTs.

The heterogeneity test of 34 included studies revealed a high level of heterogeneity (*I*^2^ = 99.5%; *p* < 0.001). A random-effects model, supplemented by subgroup analysis, was employed to explore the sources of heterogeneity. The results indicated that the overall prevalence of SWMM was 47% (95% CI, 40%–54%) (see Figure 2). The results of the Egger test demonstrated a significant difference (*p* = 0.021). The scatter distribution in the funnel plot was asymmetrical, indicating a clear presence of publication bias (see additional file S6). To further assess the potential impact of missing studies on the overall results, we applied the trim-and-fill method using the “metatrim” command. However, the analysis revealed that no studies required trimming or filling, and the random-effects pooled estimate remained unchanged (0.469; 95% CI: 0.397–0.542). Additional file S7 presents the results of the sensitivity analysis. We found no significant study effect when estimating the incidence of SWMM, suggesting that no individual study had a substantial impact on the overall results.

#### 3.4.2 Subgroup prevalence

The results of the subgroup analysis are as follows (see additional file S8): (1) When combining all studies that included infants up to 31 weeks GA (ranging from 22 to 31 weeks), the pooled prevalence of SWMM was 47% (95% CI, 40%–54%). (2) In studies limited to infants <29 weeks GA (typically 22–28 weeks), prevalence ranged from 23% (95% CI, 0%–46%) to 44% (95% CI, 26%–61%), while studies examining the broader 22–31 weeks GA range (and not excluding <29 weeks cases) showed higher prevalence at 67% (95% CI, 62%–72%). (3) In terms of the study period, studies conducted entirely after 2010 reported the highest prevalence at 56% (95% CI, 46%–66%), whereas those completed before 2010 reported a prevalence of 43% (95% CI, 28%–58%). Studies that spanned the year 2010 (i.e., with data collection overlapping this time point) demonstrated the lowest prevalence at 31% (95% CI, 23%–39%). (4) Concerning the definition of SWMM, the highest prevalence was observed at 57% (95% CI, 44%–70%) when Definition A was utilized, followed by Definition B, which yielded a prevalence of 40% (95% CI, 33%–47%). (5) With respect to sample size, studies with sample sizes between 500 and 2,000 reported a prevalence of 49% (95% CI, 36%–63%), and those with sample sizes greater than 2,000 reported a prevalence of 47% (95% CI, 36%–57%). Studies with sample sizes of less than 500 exhibited a prevalence of 43% (95% CI, 23%–62%). (6) In terms of study region, the highest prevalence of SWMM was found in China (52%, 95% CI, 34%–70%) and European countries (52%, 95% CI, 30%–73%), followed by North American countries, where the prevalence was 39% (95% CI, 29%–49%), and other countries, which reported a prevalence of 40% (95% CI, 18%–62%). (7) Regarding the denominator used to calculate the rate of SWMM incidence, the prevalence was 51% (95% CI, 42%–60%) for studies using the total population as the denominator, compared to 37% (95% CI, 29%–45%) for those using survivors as the denominator. Additionally, the prediction intervals calculated for each subgroup analysis were broadly consistent with the 95% confidence intervals reported above, further supporting the stability of these prevalence estimates.

#### 3.4.3 Heterogeneity and meta-regression

Univariable meta-regression analyses assessing gestational age range, study period, definition of SWMM, sample size, study region, and denominator of SWMM rate calculation revealed substantial and persistent residual heterogeneity (*I*^2^ = 99.6%, 99.9%, 99.9%, 99.8%, 99.9% and 99.9%, respectively). We further conducted a multivariable meta-regression incorporating all these six covariates, which included data from 34 studies. The model showed a significant joint effect of the covariates [F (6,27) = 12.85, *p* < 0.001] and explained 68.85% of the between-study variance (adjusted R-squared). Among the variables, gestational age range and definition of SWMM were statistically significant (gestational age range: coefficient = 0.356, *p* < 0.001; definition of SWMM: coefficient = −0.149, *p* = 0.006). The other covariates—study region, study period, sample size, and denominator of SWMM calculation—did not reach statistical significance (*p* > 0.05). Despite the model's explanatory power, residual heterogeneity remained extremely high (*I*^2^_res = 99.30%), indicating that a large proportion of between-study variability remains unexplained.

#### 3.4.4 Risk factors

Twelve studies investigated 25 risk factors for SWMM via multivariate models (see Table 1). These variables were categorized into four major groups: antenatal factors (24%, 6/25), perinatal factors (44%, 11/25), neonatal factors (24%, 6/25), and other factors (8%, 2/25). Four variables were identified as definitive risk factors for SWMM in very preterm infants (VPIs), based on either all low- and moderate-risk studies demonstrating a positive association (if at least three studies) or the majority of low- and moderate-risk studies showing a positive association (if at least five studies). The identified risk factors included gestational age, birth weight, male sex, and patent ductus arteriosus (PDA). Two variables—air leaks and duration of mechanical ventilation—were considered likely associated with SWMM. Fifteen variables that yielded conflicting results in studies with low and moderate risk of bias, or were positive in only one study, were deemed to have an unclear association with SWMM (refer to Table 1 for specific variables). Additionally, chorioamnionitis, maternal age, small for gestational age (SGA) status, and body temperature were classified as non-risk factors.

**Figure 3:**
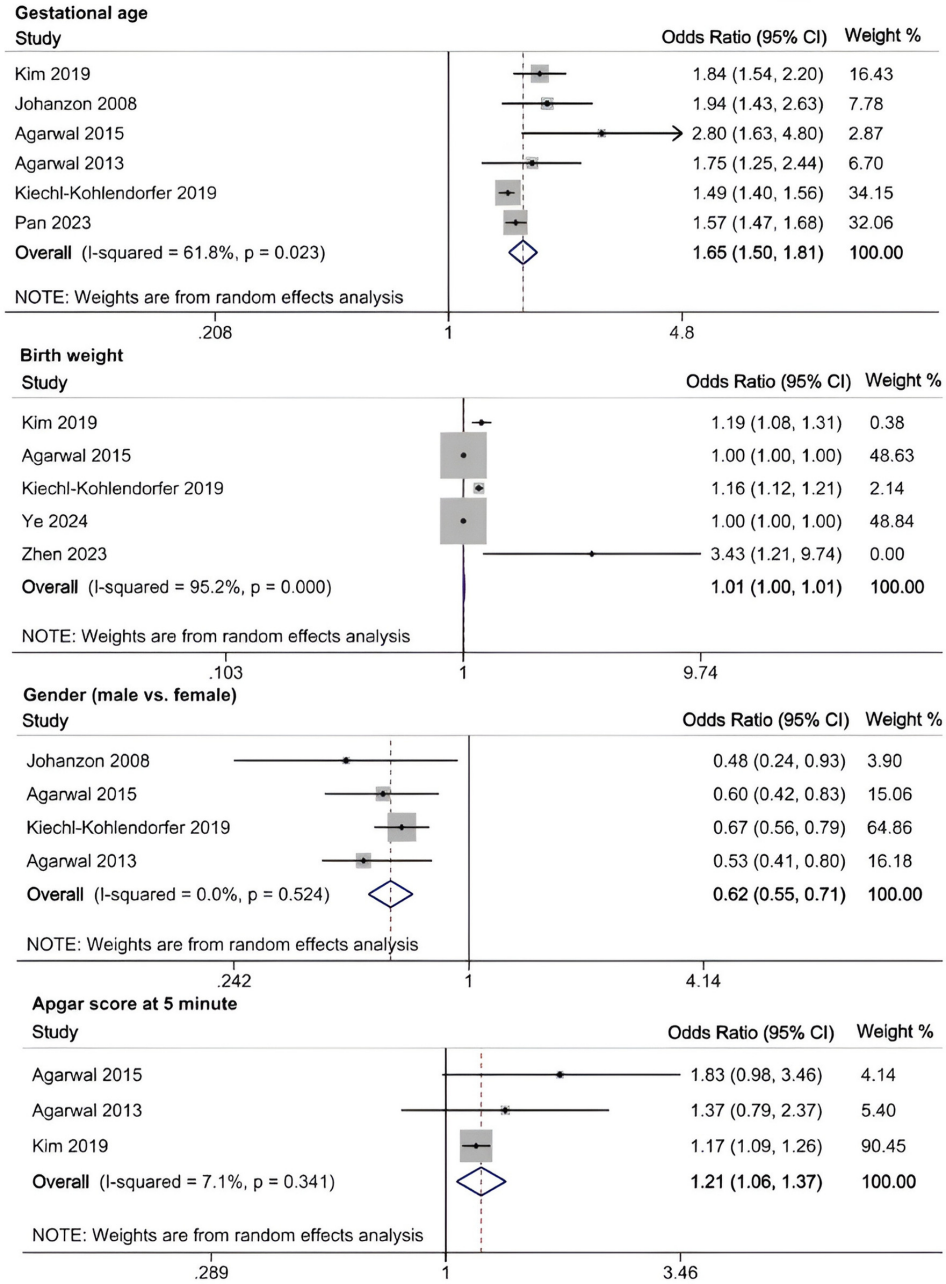
Meta-analysis of perinatal factors related to SWMM in VPTs.

**Figure 4:**
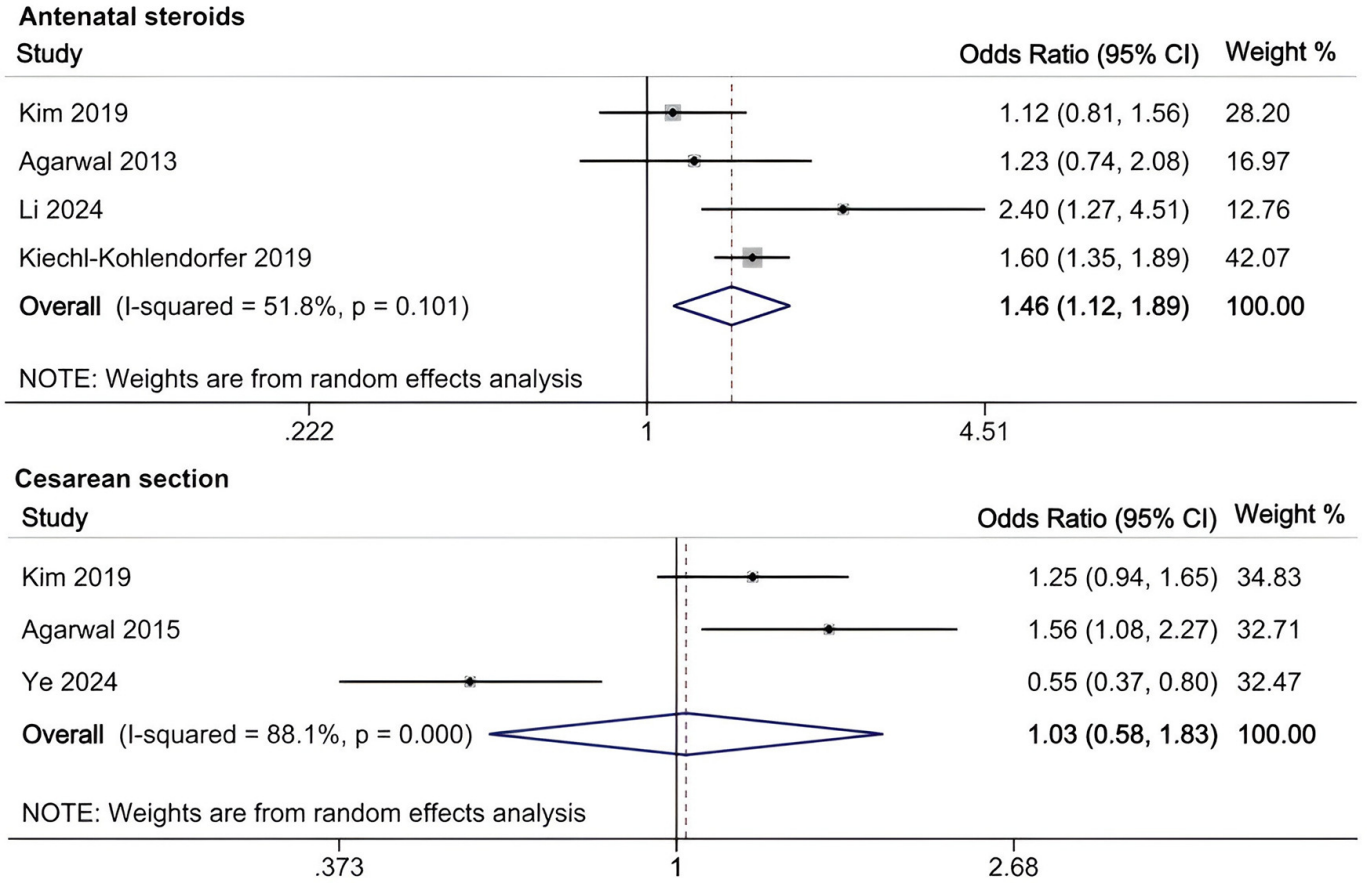
Meta-analysis of antenatal factors related to SWMM in VPTs.

**Figure 5:**
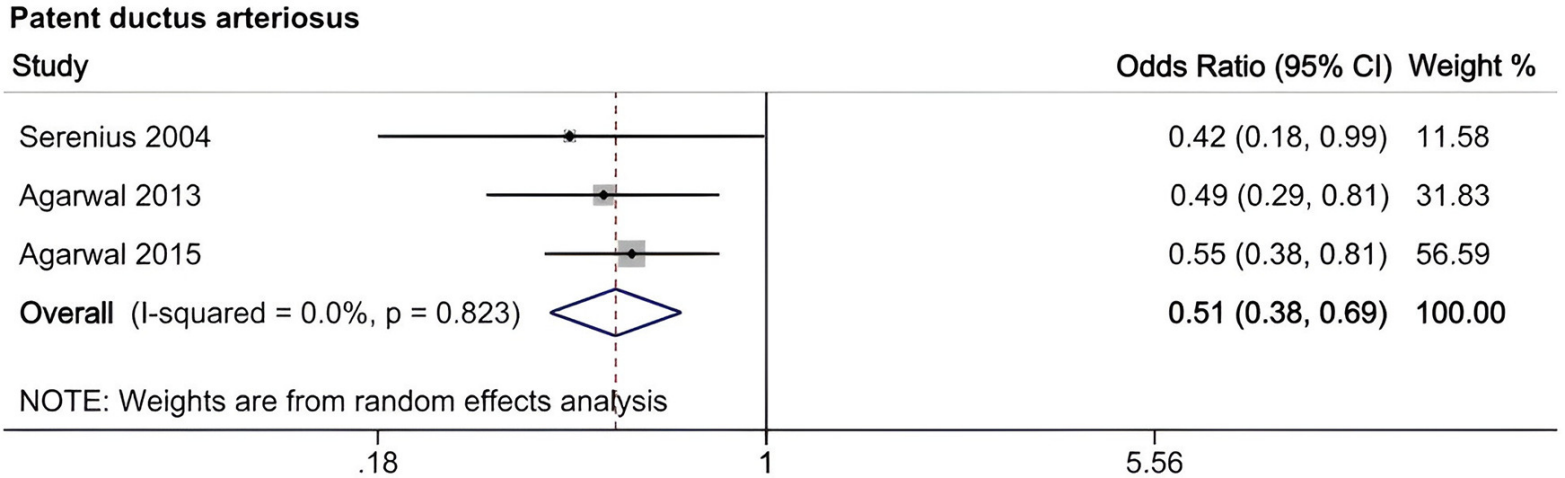
Meta-analysis of neonatal factors related to SWMM in VPTs.

A meta-analysis was conducted to evaluate risk factors, utilizing at least three low- or moderate-risk-of-bias studies that demonstrated consistent definitions and reference ranges for the risk factors (Figures 3–5). The pooled analysis identified seven potential risk factors associated with SWMM in very preterm infants: gestational age, male sex, birth weight, antenatal steroids, cesarean section, 5-minute Apgar score, and patent ductus arteriosus (PDA). Among these factors, the associations of SWMM with birth weight and cesarean section did not achieve statistical significance. The results of the risk factor analysis are summarized in Table 2. Later gestational age (OR: 1.65; 95% CI, 1.50–1.81), a higher 5 min Apgar score (OR: 1.21; 95% CI, 1.06–1.37), and antenatal steroid treatment (OR: 1.46; 95% CI, 1.12–1.89) were associated with an increased risk of SWMM. Conversely, male gender (OR: 0.62; 95% CI, 0.55–0.71) and significant PDA (OR: 0.51; 95% CI, 0.38–0.69) were linked to a reduced risk of SWMM. Due to the limited number of studies included (*n* < 6) for each specific risk factor, an assessment of publication bias using funnel plots or statistical tests (e.g., Egger's test) was not performed, as these methods require a minimum of ten studies for reliable interpretation.

**Table 2:** Significant risk factors for SWMM in very preterm infants.

No.	Risk factors	Number of included studies	OR	95% CI	*I* ^2^	*P*-value
1	Gestational age	6	1.65	1.50–1.81	61.80%	0.023
2	Gender (male)	4	0.62	0.55–0.71	0.00%	0.524
3	Birth weight	5	1.01	1.00–1.01	95.20%	<0.001
4	Antenatal steroids	4	1.46	1.12–1.89	51.80%	0.101
5	Cesarean section	3	1.03	0.58–1.83	88.10%	<0.001
6	5 min Apgar score	3	1.21	1.06–1.37	7.10%	0.341
7	PDA	3	0.51	0.38–0.69	0.00%	0.823

## 4 Discussion

Our meta-analysis of 34 studies (*n* = 156,739) revealed an overall survival without major morbidities (SWMM) incidence of 47% (95% CI, 40%–54%). In very preterm infants, incidence varied significantly by gestational age (GA): 67% (95% CI, 62%–72%) for GA <32 weeks, compared to 23%–44% for GA <29 weeks (95% CI, 0%–61%). The comparable rates between GA <28 weeks (44%) and GA <29 weeks (35%) may reflect international variations in resuscitation practices and family decision-making.

Temporal analysis revealed an increase in post-2010 rates of SWMM to 56% (95% CI, 46%–66%), potentially reflecting advancements in medical technology and changes in healthcare policy. However, contradictory trends emerged within GA-specific subgroups: the post-2010 SWMM rate was 33% (95% CI, 21%–45%) for infants born at less than 30 weeks of gestation, while those born at less than 32 weeks exhibited an estimated SWMM prevalence of 67% (95% CI, 61%–73%). This underscores the necessity for multivariable analyses that incorporate national-level characteristics. International comparisons revealed substantial disparities: the SWMM prevalence in China was 11% (95% CI, 9%–13%), compared to 45% (95% CI, 25%–66%) in Europe for infants born at less than 30 weeks of gestation, a difference that exceeds previous multinational observations (9). These variations are likely influenced by economic factors, healthcare policies, and sociocultural determinants. Conversely, very preterm infants (GA <32 weeks) demonstrated a narrower international variation in SWMM prevalence (66%–73%), which aligns with existing evidence (9).

Mechanistically, the determinants of SWMM operate through three interrelated pathways: (1) biological maturity (gestational age-dependent organ development), (2) therapeutic interventions (antenatal corticosteroids and patent ductus arteriosus management), and (3) socioclinical factors (sex disparities and denominator selection). Our findings underscore the hierarchical significance of biological maturity, as each additional week of gestational age increased the probability of SWMM by 65% (odds ratio 1.65; 95% confidence interval, 1.50–1.81), which aligns with the existing fetal physiology literature (58).

The differential diagnostic criteria significantly influenced the prevalence estimates of SWMM. Definition A, which exclusively excludes septic complications, demonstrated an estimated SWMM rate of 57% (95% CI, 44%–70%), compared to 40% (95% CI, 33%–47%) in the group defined by Definition B. This discrepancy may be attributed to the more lenient inclusion criteria of Definition B. Counterintuitively, studies utilizing total cohort denominators (including deceased infants) reported an estimated SWMM prevalence of 51% (95% CI, 42%–60%), whereas those using survivor-only denominators reported a prevalence of 37% (95% CI, 29%–45%). This paradox may arise from variations in regional mortality rates; for instance, higher mortality in the cohort of infants born at less than 26 weeks gestational age automatically elevates the SWMM rates based on total denominators. Therefore, standardizing both SWMM definitions and calculation methods is critical for valid cross-national comparisons.

Our analysis identified several protective factors for SWMM. Very preterm infants with a later gestational age are more likely to survive without major morbidity. A later gestational age contributes to the maturation of organ systems, which, in turn, increases postnatal resilience. Specifically, for infants born before 27 weeks of gestation, neonatal survival rates improve by 2% for each additional day spent *in utero* (59). This correlation is also observed in very preterm infants who are small for their gestational age (60). Although tocolysis primarily aims to delay rather than prevent preterm birth, prolonged intrauterine development through pregnancy maintenance may enhance SWMM outcomes (61). The administration of antenatal steroids has demonstrated protective effects (OR: 1.46; 95% CI, 1.12–1.89), potentially through multiple mechanisms: reduced risk of intracranial hemorrhage and periventricular leukomalacia (61), increased pulmonary maturation, and decreased perinatal mortality (62). A meta-analysis of clinical trials has confirmed the effectiveness of antenatal corticosteroids when administered to mothers one week before delivery, resulting in reduced neonatal mortality and the incidence and severity of bronchopulmonary dysplasia (BPD), intracranial hemorrhage, retinopathy of prematurity (ROP) and necrotizing enterocolitis (NEC) (63). However, their neurological benefits appear limited in multifetal gestations (64). In this study, the 5 min Apgar score demonstrated a significant positive correlation with SWMM, highlighting underscoring clinical relevance in predicting neonatal outcomes. As a well-established and commonly used assessment measure in the field of neonatology, the 5 min Apgar score has long been recognized for its traditional and significant role in predicting long-term outcomes in term infants, as clearly demonstrated by numerous high-quality studies (24, 48). However, its role in predicting long-term outcomes for very preterm populations is limited constrained high interobserver variability and the confounding effects of intensive care exposure (65). Further research is needed to develop more accurate and reliable prognostic tools specifically tailored to this unique patient population.

Male sex significantly reduced the probability of SWMM (OR: 0.62; 95% CI, 0.55–0.71), which aligns with established sex-specific vulnerability patterns in preterm infants (66). Compared to females, male very preterm infants (VPIs) are more likely to experience death or major morbidity. This disparity may be partially attributed to the heightened sensitivity of male preterm neonates to oxidative stress and the neurodevelopmental impacts associated with postnatal growth (67, 68). Persistent Patent Ductus Arteriosus (PDA), which is present in 50% of infants born at less than 32 weeks of gestational age, significantly reduced the probability of SWMM (OR: 0.51; 95% CI, 0.38–0.69) and was associated with a sixfold increase in mortality and respiratory morbidity (69, 70). Although causality remains unproven, early pharmacological or surgical closure of PDA (for infants weighing less than 4 kg) may improve respiratory outcomes despite the associated procedural risks (69). Implementing strategies to manage PDA in very preterm infants may enhance the likelihood of favorable SWMM outcomes.

In the meta-analysis of this study, the association between the rate of SWMM and birth weight did not reach statistical significance. Given the potential collinearity between gestational age and birth weight in perinatal research (71), this finding further suggests that the SWMM rate may be more appropriately explored in relation to gestational age rather than birth weight. Agarwal et al. reported a positive correlation between cesarean section and SWMM, while Ye reported an inverse relationship (28, 53). It is anticipated that cesarean sections can increase maternal and neonatal morbidity to some extent. Research indicates that maternal and perinatal outcomes improve when the cesarean section rate remains below 10% (72). Respiratory depression at birth, an Apgar score of less than 3, and hypoxic-ischemic encephalopathy (HIE) are more frequently observed in patients who undergo cesarean sections, particularly in emergency situations (73). This may be attributed to a higher proportion of high-risk mothers experiencing fetal distress and delayed referrals in the emergency cesarean section group. To further investigate the relationship between cesarean sections and SWMM in VPIs, a detailed categorization of cesarean sections is essential. Additionally, regional variations in cesarean section rates must be taken into account. This study revealed that air leaks and the duration of mechanical ventilation are likely associated with SWMM in VPIs. Research has indicated that air leaks are linked to various morbidities (74). In infants experiencing pulmonary air leaks, oxygen saturation levels can fluctuate significantly, often necessitating high fractions of inspired oxygen (FiO2) and prolonged supplemental oxygen, which may increase the risk of ROP (75). Additionally, pulmonary air leaks can lead to alveolar collapse, lung inflammation, and injury, requiring ventilator support and oxygen supplementation. These complications may result in barotrauma, oxygen toxicity, and BPD (76). Air leaks can also induce hypoxia, hypercapnia, and hypotension, disrupting cerebral blood flow and resulting in IVH (77). Prolonged mechanical ventilation is associated with an increased risk of BPD and subsequent neurodevelopmental impairments (78, 79). These likely and the remaining unclear associated risk factors require further studies to verify their relationship with SWMM in VPIs.

## 5 Strengths and limitations

A fundamental strength of the current analysis is the adoption of a robust methodology. A comprehensive literature search was conducted across eight electronic databases, encompassing publications in both English and Chinese. Notably, this study is the first to provide an estimate of the pooled prevalence of SWMM in VPIs, as well as a systematic assessment of the risk factors associated with this condition. Our findings offer valuable insights for neonatologists, families of VPIs, public health managers, and researchers. By elucidating the prevalence of SWMM in VPIs and the associated risk factors, our study provides actionable guidance for clinical practice and family counseling. Clinicians should prioritize quality improvement initiatives by integrating the identified modifiable risk factors into standardized care protocols. For parents, the observed moderate prevalence rates of SWMM may offer reassurance regarding their infants' developmental prospects, thereby alleviating anxiety related to prematurity. However, potential limitations of the present work should be acknowledged. First, there was considerable heterogeneity among the included studies, possibly due to the combined influences of various factors, including differences in gestational age groups, divergent definitions of SWMM, and inconsistencies in the denominators used for calculating the SWMM rate. Second, an in-depth analysis of certain risk factors for SWMM was not feasible because they were either not reported in the original research or only a limited number of risk factors were addressed. Third, the systematic review did not include unpublished articles or studies that did not adhere to definitions A or B of SWMM, and studies with negative results may have been omitted, likely contributing to publication bias. Finally, the exclusion of works published in other languages limited the comprehensiveness of the included literature. Future studies are needed to address these limitations and investigate the risk factors for SWMM in VPIs in a more thorough manner.

## 6 Conclusion

This systematic review reports a 47% rate of SWMM among very preterm infants, with rates varying significantly by gestational age. Later gestational age, higher 5 min Apgar scores, and antenatal steroid use are independently associated with improved SWMM rates, while persistent PDA and male sex are linked to reduced SWMM rates. Clinical implications include the optimization of prenatal steroid protocols and the intensification of PDA management to enhance outcomes. However, the heterogeneity in SWMM definitions and regional variations in neonatal practices limit the generalizability of these findings. Future efforts should prioritize standardized outcome criteria, multicenter prospective studies, and interventions targeting identified risk factors to improve outcomes in this high-risk population.

## Data Availability

The original contributions presented in the study are included in the article/Supplementary Material, further inquiries can be directed to the corresponding authors.
