# Supplementary material for: Prevalence of survival without major morbidity and associated risk factors among very preterm infants: a systematic review and meta-analysis
Source: Front Pediatr. 2025 Aug 18;13:1628472. doi: 10.3389/fped.2025.1628472 (PMC12400820; doi:10.3389/fped.2025.1628472)
Supplement: Supplementary file 1 [file Supplementaryfile1.docx]

Supplementary Material

**Contents of** **Additional File (DOC (Microsoft Word):**

Additional File S1. Search strategies.

Additional File S2. Eligibility criteria during selection.

Additional File S3. Criteria for grading methodological quality.

Additional File S4. Quality assessment of included studies.

Additional File S5. Defining the strength of a risk factor.

Additional File S6. Funnel plot.

Additional File S7. Sensitivity analysis.

Additional File S8. Subgroup analyses of the prevalence of SWMM in infants with GA<32wk.

Additional File S9. Definition of major morbidity in included studies.

Additional File S10. Excluded full-text articles with reasons.

**Additional File S1. Search strategies**

**PubMed**

**Search Query**

**#1** "Infant, premature" [MeSH] OR "premature birth" [MeSH] OR "Infant, extremely low birth weight"[MeSH] OR "preterm infants"[Title/Abstract] OR "preterm infant"[Title/Abstract] OR "premature infant"[Title/Abstract] OR "premature infants"[Title/Abstract] OR "very preterm infants"[Title/Abstract] OR "extremely preterm infants"[Title/Abstract]

**#2** "morbidity-free survival"[Title/Abstract] OR "major morbidity"[Title/Abstract] OR "major morbidities"[Title/Abstract] OR "severe complications"[Title/Abstract] OR "severe morbidity"[Title/Abstract] OR "major complications"[Title/Abstract]

**#3**"risk factors"[MeSH] OR "risk factors"[Title/abstract] OR "risk factor"[Title/abstract] OR "risk scores"[Title/abstract] OR "risk score"[Title/abstract]

**#4 #**1 AND #2 AND #3

**Embase**

**No. Query**

**#1** 'prematurity'/exp OR 'very preterm infant'/exp OR 'very preterm infants':ti,ab,kw OR 'extremely preterm infants'/exp OR 'extremely preterm infants':ti,ab,kw

**#2** 'newborn morbidity'/exp OR 'survival without major morbidity':ti,ab,kw OR 'survival without major morbidities':ti,ab,kw OR 'survival without serious complications':ti,ab,kw OR 'survival free of major complications':ti,ab,kw OR 'survival without severe complications':ti,ab,kw OR 'morbidity-free survival':ti,ab,kw

**#3** 'risk factor'/exp OR 'risk factor':ti,ab,kw OR 'risk score':ti,ab,kw **#4** newborn:ti,ab,kw OR baby:ti,ab,kw OR prematurity:ti,ab,kw OR 'low birth weight':ti,ab,kw OR infant:ti,ab,kw

**#4 #**1 AND #2 AND #3

**Scopus**

**No. Query**

#1 TITLE-ABS-KEY ("premature birth" OR "preterm infant" OR "premature infant" OR "very preterm infant" OR "extremely preterm infant" OR "extremely low birth weight" OR "very low birth weight")

#2 TITLE-ABS-KEY (“morbidity-free survival" OR " major morbidity" OR "major morbidities" OR "severe complications" OR "severe morbidity" OR "major complications")

#3 TITLE-ABS-KEY ("risk factor" OR "risk score")

#4 **#**1 AND #2 AND #3

**Cochrane Library**

**ID Search**

**#1** "premature birth" OR "preterm infant" OR "premature infant" OR "very preterm infant" OR "extremely preterm infant" OR "extremely low birth weight" OR "very low birth weight" in Title Abstract Keyword

**#2** "major morbidity" OR "serious complications" OR "major morbidities" OR "morbidity-free survival" OR "severe morbidity" OR "severe complications" in Title Abstract Keyword

**#3** "risk factor" OR "risk score" in Title Abstract Keyword - (Word variations have been searched)

**#4** #1 AND #2 AND #3

**Web of Science**

**ID Search**

**#1** TS= ("premature birth" OR "preterm infant" OR "premature infant" OR "very preterm infant" OR "extremely preterm infant" OR "extremely low birth weight" OR "very low birth weight")

**#2** TS= (serious complications) OR TS= (major morbidity) OR TS= (major morbidities) OR TS= (morbidity-free survival) OR TS= (severe morbidity) OR TS= (severe complications) OR TS= (survival free of major complications)

**#3** TS= ("risk factor" OR "risk score")

**#4** #1 AND #2 AND #3

**China National Knowledge Infrastructure (CNKI) - field searching in Chinese**

**序号 检索表达式**

**#1** 主题：极早产儿+超早产儿（精确)

**#2** 主题：严重并发症（精确）

**#3** 主题：影响因素+危险因素（精确）

**#4** #1 AND #2 AND #3

**Wanfang Datebase - field searching in Chinese**

**序号 检索表达式**

**#1** 主题：(极早产儿 or 超早产儿）

**#2** 主题：(严重并发症)

**#3** 主题：(影响因素 or 危险因素)

**#4** #1 AND #2 AND #3

**the Chinese Biomedical Database (CBM) - field searching in Chinese**

**序号 检索表达式**

**#1** 主题：(婴儿，早产儿）

**#2** 主题：(严重并发症)

**#3** 主题：(影响因素 or 危险因素)

**#4** #1 AND #2 AND #3

**Additional File S2. Eligibility criteria during selection**

**Additional File S2 -A Eligibility Criteria**

| **1.** | **Study design** | Cross-sectional studies or cohort studies |
| --- | --- | --- |
| **2.** | **Study participants** | very preterm infants including extremely preterm infants, with a gestational age <32 weeks |
| **3.** | **Definition of major morbidity** | - Chronic lung disease (CLD)/ bronchopulmonary dysplasia (BPD) - Severe neurological injury (SNI) - Necrotizing enterocolitis (NEC) - Severe retinopathy of prematurity (ROP) - Sepsis |
| **4.** | **Outcome indicators** | The rate of SWMM, risk factors associated with SWMM or DOMM |
| **5.** | **Sample size** | Greater than 100 |

**Additional File S2 -B Definition of major morbidity**

|  | **Major morbidity** | **Definitions** |
| --- | --- | --- |
| **1.** | Chronic lung disease (CLD)/ bronchopulmonary dysplasia (BPD) | Receiving any supplemental oxygen at 36 weeks of age or at time of transfer of discharge. |
| **2.** | Severe neurological injury (SNI) | Intraventricular hemorrhage grade 3 or greater by the Papile classification, or periventricular leukomalacia or persistent parenchymal echogenicity. |
| **3.** | Necrotizing enterocolitis (NEC) | Stage 2 or greater per modified Bell criteria. |
| **4.** | Severe retinopathy of prematurity (ROP) | Stage 3 or greater by international staging criteria or requiring laser or intravitreal injection in at least one eye. |
| **5.** | Sepsis | Positive blood or cerebrospinal fluid culture and antibiotic therapy or intent of antibiotics therapy ≥5 days. |

**Additional File S3. Criteria for grading methodological quality**

| **Assessment quality of cross-sectional studies with the AHRQ Criteria** | |
| --- | --- |
| Low risk of bias | The study meets high-quality standards in all key domains (e.g., design, sample representativeness, data collection methods, bias control, statistical analysis) with no significant flaws identified. |
| Some concerns | The study raises potential issues in at least one key domain (e.g., selection bias, unclear measurement methods, inadequate statistical analysis), but the overall results remain moderately credible. |
| High risk of bias | The study exhibits critical flaws in at least one key domain (e.g., poor sample representativeness, unscientific data collection, failure to control key confounding factors), or multiple concerns substantially undermine the credibility of the findings. |
| **Assessment quality of observational studies with the Newcastle-Ottawa Scales (NOS)** | |
| Low risk of bias | Up to one item was judged inadequate in a study. |
| Medium risk of bias | Up to three items was judged inadequate in a study. |
| High risk of bias | More than three items were judged inadequate in a study. |
| Very high risk of bias | There was no description of methods. |

**Additional File S4. Defining the strength of a risk factor.**

| Definite |
| --- |
| All low and moderate risk of bias studies positive (at least three studies) |
| Majority (more than 50%) low and moderate risk of bias studies positive (at least five studies) |
| Likely |
| All low and moderate risk of bias studies positive (two studies) |
| Majority (more than 50%) low and moderate risk of bias studies positive (2-4 studies) |
| Unclear |
| All low and moderate risk of bias studies positive (one study) |
| Low and moderate risk of bias studies show mixed or conflicting results |
| A majority (more than 50%) of studies negative but at least one low or moderate risk of bias study positive |
| Not a risk factor |
| No low or moderate risk of bias studies positive |

**Additional File S5.**

**Table A Quality assessment of including cross-sectional studies (AHRQ)**

| References | 1) Define the source of information (survey, record review) | 2) List inclusion and exclusion criteria for exposed and unexposed subjects (cases and controls) or refer to previous publications | 3) Indicate time period used for identifying patients | 4) Indicate whether or not subjects were consecutive if not population-based | 5) Indicate if evaluators of subjective components of study were masked to other aspects of the status of the participants | 6) Describe any assessments undertaken for quality assurance purposes | 7) Explain any patient exclusions from analysis | 8) Describe how confounding was assessed and/or controlled. | | 9) If applicable, explain how missing data were handled in the analysis | | 10) Summarize patient response rates and completeness of data collection | 11) Clarify what follow-up, if any, was expected and the percentage of patients for which incomplete data or follow-up was obtained | total quality score |
| --- | --- | --- | --- | --- | --- | --- | --- | --- | --- | --- | --- | --- | --- | --- |
| Jiang 2024 | 1 | 1 | 1 | 1 | 0 | 0 | 1 | 1 | 1 | | 1 | | 1 | 9 |
| Kavurt 2023 | 1 | 1 | 1 | 1 | 0 | 0 | 0 | 1 | 0 | | 1 | | 1 | 7 |

**Table B Quality assessment of including cohort studies (NOS)**

| References | Selection | | | | Comparability | Outcomes | | | Total (max score: 9) |
| --- | --- | --- | --- | --- | --- | --- | --- | --- | --- |
|  | Representative-ness of the exposed cohort | Selection of the non-exposed cohort | Ascertainment of exposure to implants | Demonstration  that outcome of interest was not present at start of study | Comparability  of cohorts on the basis of the design or analysis | Assessment of outcome | Was follow up long enough for outcomes to occur | Adequacy of follow up of cohorts |  |
| Agarwal 2015 | ★ | ★ | ★ | ★ | ★ | ★ | ★ | ★ | 8 |
| Agarwal 2013 | ★ | ★ | ★ | ★ | ★ | ★ | ★ | ★ | 8 |
| Ancel 2015 | ★ | ★ | ★ | ★ | ★ | ★ | ★ | ★ | 8 |
| Anderson 2016 | Selected group | ★ | ★ | ★ | ★ | ★ | ★ | ★ | 7 |
| Beltempo 2019 | ★ | ★ | ★ | ★ | Not described | ★ | ★ | ★ | 7 |
| Cao 2021 | ★ | ★ | ★ | ★ | ★ | ★ | ★ | ★ | 8 |
| Chang 2018 | ★ | ★ | ★ | ★ | ★ | ★ | ★ | ★ | 8 |
| Costeloe 2016 | ★ | ★ | ★ | ★ | ★ | ★ | ★ | ★ | 8 |
| Cust 2003 | ★ | ★ | ★ | ★ | ★ | ★ | ★ | ★ | 8 |
| Higgins 2024 | ★ | ★ | Not described | ★ | ★ | ★ | ★ | ★ | 7 |
| Isayama 2012 | ★ | ★ | ★ | ★ | ★ | ★ | ★ | ★ | 8 |
| Jiang 2020 | ★ | ★ | ★ | ★ | ★ | ★ | ★ | ★ | 8 |
| Johanzon 2008 | ★ | ★ | ★ | ★ | ★ | ★ | ★ | ★ | 8 |
| Kiechl-Kohlendorfer 2019 | ★ | ★ | ★ | ★ | ★ | ★ | ★ | ★ | 8 |
| Kim 2019 | ★ | ★ | ★ | ★ | ★ | ★ | ★ | ★ | 8 |
| Kong 2016 | ★ | ★ | ★ | ★ | ★ | ★ | ★ | ★ | 8 |
| Li 2024 | ★ | ★ | ★ | ★ | Not described | ★ | ★ | ★ | 7 |
| Li 2024 | ★ | ★ | ★ | ★ | ★ | ★ | ★ | Not described | 7 |
| Marlow 2015 | ★ | ★ | ★ | ★ | Not described | ★ | ★ | ★ | 7 |
| Morgillo 2014 | ★ | ★ | ★ | ★ | ★ | ★ | ★ | ★ | 8 |
| Nourkami-Tutdibi 2021 | ★ | ★ | ★ | ★ | Not described | ★ | ★ | ★ | 7 |
| Pan 2023 | ★ | ★ | ★ | ★ | ★ | ★ | ★ | ★ | 8 |
| Peng 2022 | ★ | ★ | ★ | ★ | ★ | ★ | ★ | ★ | 8 |
| Serenius 2004 | ★ | ★ | ★ | ★ | ★ | ★ | ★ | ★ | 8 |
| Simic 2014 | ★ | ★ | ★ | ★ | ★ | ★ | ★ | ★ | 8 |
| Stoll 2010 | ★ | ★ | ★ | ★ | ★ | ★ | ★ | ★ | 8 |
| Stoll 2015 | ★ | ★ | ★ | ★ | ★ | ★ | ★ | ★ | 8 |
| Wu 2021 | ★ | ★ | ★ | ★ | ★ | ★ | ★ | ★ | 8 |
| Ye 2024 | ★ | ★ | ★ | ★ | ★ | ★ | ★ | ★ | 8 |
| Yeung 2024 | ★ | ★ | ★ | ★ | Not described | ★ | ★ | Not described | 6 |
| Zayek 2011 | ★ | ★ | ★ | ★ | ★ | ★ | ★ | Not described | 7 |
| Zhen 2023 | ★ | ★ | ★ | ★ | Not described | ★ | ★ | ★ | 7 |
| Zhu 2021 | ★ | ★ | ★ | ★ | ★ | ★ | ★ | ★ | 8 |

★ Meet the scoring conditions

**Additional File S6.**

**A Funnel plot of the prevalence of SWMM in VPTs**

**
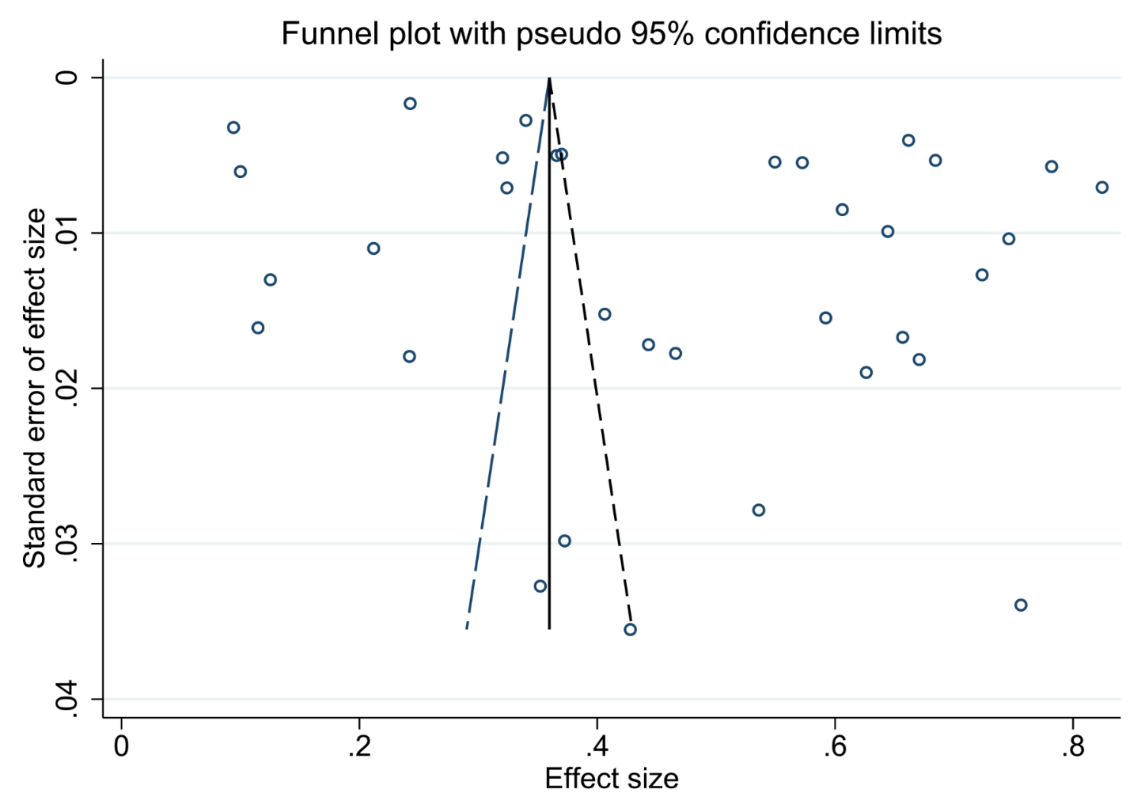
**

**Funnel plot after trim-and-fill analysis (no imputed studies added)**

**Additional File S7. Sensitivity analysis of the prevalence of SWMM in VPTs**

**
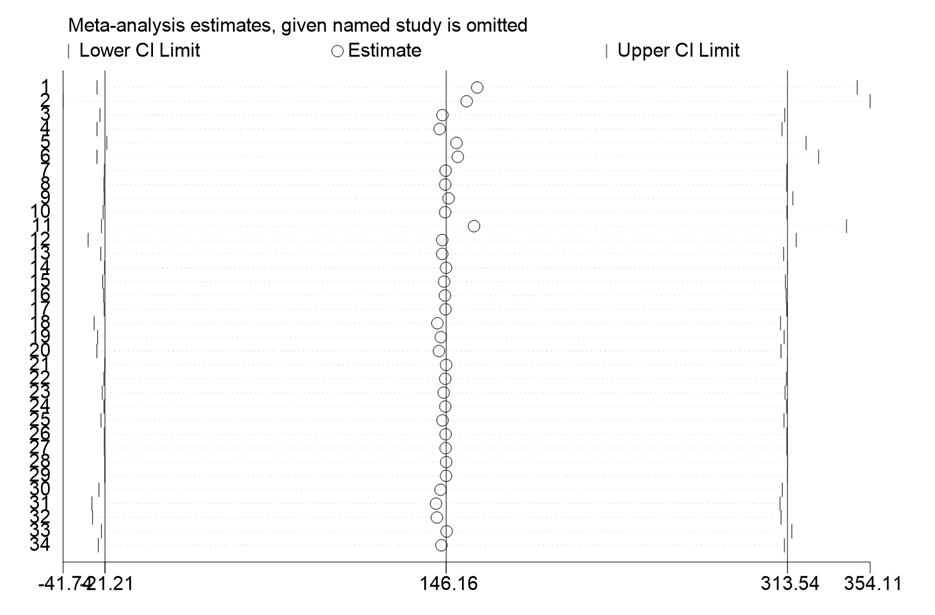
**

**Additional File S8. Subgroup analyses of the prevalence of SWMM in infants with GA<32wk.**

| **Group standard** | | **Combined effect size** | | | | | | |  | | **Heterogeneity test (total)** | | | | |  |  |  |
| --- | --- | --- | --- | --- | --- | --- | --- | --- | --- | --- | --- | --- | --- | --- | --- | --- | --- | --- |
|  |  | **No. studies** | **Prevalence rate, % (95%CI)** | | | | **95% prediction interval** | | | | | | ***P*** | | ***I^2^*, %** | | | |
|  |  |  | **total** | **GA<30wk** | **GA<32wk** | **total** | | **GA<30wk** | | **GA<32wk** | |  | |  | | | |  |
| The total result | | 34 | 0.47 (0.40, 0.54) |  |  | 0.47 (0.40, 0.55) | |  | |  | | <0.001 | | 99.9 | | |  |  |
| Gestational age (Max GA Range)☆ | |  |  |  |  |  | |  | |  | |  | |  | | |  |  |
|  | <26 wk | 2 | 0.23 (0.00, 0.46) |  |  | 0.19 (0.16, 0.22) | |  | |  | | <0.001 | | 97.6 | | |  |  |
|  | <27 wk | 7 | 0.30 (0.18, 0.41) |  |  | 0.30 (0.18, 0.41) | |  | |  | | <0.001 | | 99.3 | | |  |  |
|  | <28 wk | 5 | 0.44 (0.26, 0.61) |  |  | 0.44 (0.26, 0.61) | |  | |  | | <0.001 | | 99.8 | | |  |  |
|  | <29 wk | 7 | 0.35 (0.30, 0.40) |  |  | 0.35 (0.30, 0.40) | |  | |  | | <0.001 | | 99.7 | | |  |  |
|  | <32 wk | 13 | 0.67 (0.62, 0.72) |  |  | 0.67 (0.62, 0.72) | |  | |  | | <0.001 | | 99.3 | | |  |  |
| Study period | |  |  |  |  |  | |  | |  | |  | |  | | |  |  |
|  | Before 2010 | 11 | 0.43 (0.28, 0.58) | 0.37 (0.26, 0.48) | 0.69 (0.63, 0.75) | 0.45 (0.30, 0.59) | | 0.39 (0.28, 0.50) | | 0.67 (0.66, 0.67) | | <0.001 | | 99.9 | | |  |  |
|  | Overlapping 2010 | 7 | 0.31 (0.23, 0.39) | 0.31 (0.23, 0.39) |  | 0.31 (0.23, 0.39) | | 0.31 (0.23, 0.39) | |  | | <0.001 | | 99.8 | | |  |  |
|  | After 2010 | 16 | 0.56 (0.46, 0.66) | 0.33 (0.21, 0.45) | 0.67 (0.61, 0.73) | 0.56 (0.46, 0.66) | | 0.33 (0.21, 0.45) | | 0.67 (0.61, 0.73) | | <0.001 | | 99.9 | | |  |  |
| Definition of MM | |  |  |  |  |  | |  | |  | |  | |  | | |  |  |
|  | A* | 14 | 0.57 (0.44, 0.70) | 0.45 (0.27, 0.64) | 0.72 (0.66, 0.78) | 0.58 (0.45, 0.71) | | 0.48 (0.29, 0.67) | | 0.72 (0.66, 0.78) | | <0.001 | | 99.9 | | |  |  |
|  | B^#^ | 20 | 0.40 (0.33, 0.47) | 0.27 (0.22, 0.33) | 0.63 (0.58, 0.68) | 0.40 (0.33, 0.47) | | 0.27 (0.22, 0.33) | | 0.63 (0.58, 0.68) | | <0.001 | | 99.9 | | |  |  |
| Sample size | |  |  |  |  |  | |  | |  | |  | |  | | |  |  |
|  | <500 | 6 | 0.43 (0.23, 0.62) | 0.43 (0.23, 0.62) |  | 0.46 (0.25, 0.66) | | 0.46 (0.25, 0.66) | |  | | <0.001 | | 98.8 | | |  |  |
|  | 500-2000 | 12 | 0.49 (0.36, 0.63) | 0.36 (0.23, 0.48) | 0.69 (0.64, 0.73) | 0.49 (0.36, 0.63) | | 0.36 (0.23, 0.48) | | 0.69 (0.64, 0.73) | | <0.001 | | 99.6 | | |  |  |
|  | >2000 | 16 | 0.47 (0.36, 0.57) | 0.27 (0.20, 0.34) | 0.67 (0.60, 0.73) | 0.47 (0.36, 0.57) | | 0.27 (0.20, 0.34) | | 0.67 (0.60, 0.73) | | <0.001 | | 100 | | |  |  |
| Study region | |  |  |  |  |  | |  | |  | |  | |  | | |  |  |
|  | China | 12 | 0.52 (0.34, 0.70) | 0.11 (0.09, 0.13) | 0.66 (0.58, 0.73) | 0.52 (0.34, 0.70) | | 0.11 (0.09, 0.13) | | 0.66 (0.58, 0.73) | | <0.001 | | 99.9 | | |  |  |
|  | European countries | 9 | 0.52 (0.30, 0.73) | 0.45 (0.25, 0.66) | 0.73 (0.64, 0.83) | 0.54 (0.33, 0.75) | | 0.48 (0,27, 0.69) | | 0.73 (0.72, 0.74) | | <0.001 | | 99.9 | | |  |  |
|  | North American countries | 8 | 0.39 (0.29, 0.49) | 0.35 (0.30, 0.40) | 0.66 (0.65, 0.67) | 0.39 (0.29, 0.49) | | 0.35 (0.30, 0.40) | | 0.66 (0.65, 0.67) | | <0.001 | | 99.9 | | |  |  |
|  | Others | 5 | 0.40 (0.18, 0.62) | 0.32 (0.20, 0.43) | 0.72 (0.70, 0.75) | 0.40 (0.18, 0.62) | | 0.32 (0.20, 0.43) | | 0.72 (0.70, 0.75) | | <0.001 | | 99.6 | | |  |  |
| Denominator of SWMM rate calculation | |  |  |  |  |  | |  | |  | |  | |  | | |  |  |
|  | Total or those admitted into NICU | 24 | 0.51 (0.42, 0.60) | 0.32 (0.25, 0.38) | 0.67 (0.62, 0.72) | 0.52 (0.43, 0.61) | | 0.33 (0.27, 0.39) | | 0.67 (0.62, 0.72) | | <0.001 | | 99.9 | | |  |  |
|  | Survivors | 10 | 0.37 (0.29, 0.45) | 0.37 (0.29, 0.45) |  | 0.37 (0.29, 0.45) | | 0.37 (0.29, 0.45) | |  | | <0.001 | | 99.2 | | |  |  |

Abbreviation: MM, major morbidity; SWMM, survival without major morbidity.

*A: CLD/BPD, SNI, NEC, ROP

^#^B: CLD/BPD, SNI, NEC, ROP, sepsis

☆: Gestational age groups reflect the ​maximum reported GA range of the study population in individual studies, acknowledging variable lower limits across studies. For example:

- "<28 wk" includes studies with upper limits of 22-27 weeks (e.g., 23-27 wk, 22-27 wk).
- "<29 wk" includes studies with upper limits of 22-28 weeks (e.g., 22-28 wk, 24-28 wk).

| **Additional File S9. Definition of major morbidity in included studies.** | | | |
| --- | --- | --- | --- |
| **First author** | **Year** | **Region /network** | **Description of major morbidity** |
| Agarwal et al. | 2015 | Singapore | cPVL, IVH III/IV, CLD, severe ROP and NEC ⩾ 2 A/focal intestinal perforation and blood culture-positive sepsis |
| Agarwal et al. | 2013 | Singapore | cPVL, IVH III/IV, CLD, severe ROP and NEC ⩾ 2 A/focal intestinal perforation and blood culture-positive sepsis |
| Ancel et al. | 2015 | France | grade III IVH or IPH, cPVL, severe BPD, severe ROP, severe NEC |
| Anderson et al. | 2016 | USA | grade III or IV IVH, PVL, NEC, BPD, sepsis, or ROP surgery |
| Beltempo et al. | 2019 | CNN | severe BPD; SNI; severe ROP; NEC (stage ≥2) and nosocomial infection |
| Cao et al. | 2021 | China | IVH grade ≥3 or PVL, NEC stage ≥2, sepsis, ROP stage ≥3 and BPD |
| Chang et al. | 2018 | Taiwan, China | severe IVH (≥3), PVL, CLD, NEC ≥ Stage 2A, severe ROP (≥ stage 3 or requiring therapy) and late-onset sepsis |
| Costeloe, et al. | 2012 | England | severe abnormality on cerebral ultrasonography, severe BPD, ROP stage 3 or more, or laparotomy for NEC |
| Cust et al. | 2003 | New Zealand | CLD, definite NEC, ROP stage 3 or 4, IVH grade 3 or 4, PVL, porencephalic cyst, or hydrocephalus |
| Higgins et al. | 2024 | USA | IVH grade III or IV, PVL, NEC, BPD, ROP requiring intervention, or sepsis |
| Isayama et al. | 2012 | CNN | SNI (grade 3 or 4 IVH or PVL), BPD, NEC, or ROP |
| Jiang et al. | 2024 | China | severe IVH or PVL, moderate to severe BPD, severe ROP, NEC and sepsis |
| Jiang et al. | 2020 | China | sepsis, NEC, IVH, PVL, ROP, or BPD in infants who received complete care |
| Johanzon et al. | 2008 | Sweden | IVH grade 3-4, PVL, ROP grade 3-4, BPD, or NEC |
| Kavurt et al. | 2023 | Türkiye | late-onset sepsis, >grade II IVH, NEC ≥ stage 2, severe ROP requiring treatment, and BPD |
| Kiechl-Kohlendorfer et al. | 2019 | Austria | CLD, severe NEC, severe IVH (grades 3-4), or severe ROP (grades 3-5) |
| Kim et al. | 2019 | Korea | moderate to severe BPD, severe IVH, PVL, NEC, ROP, and LOS at the time of discharge |
| Kong et al. | 2016 | China | moderate to severe BPD, severe IVH, high grade NEC, severe ROP and sepsis. |
| Li et al. | 2024 | China | NEC, BPD, severe IVH, cPVL, severe ROP, and sepsis |
| Li et al. | 2024 | China | severe SNI, NEC (grade≥2), sepsis, moderate-to-severe BPD, and severe ROP (grade ≥ 3) |
| Marlow et al. | 2015 | UK | ROP requiring retinal surgery, moderate or severe BPD, SNI or NEC managed by laparotomy |
| Morgillo, et al. | 2014 | Switzerland | PIVH ≥3, cPVL, BPD (moderate or severe), ROP stage ≥3, NEC stage ≥2 |
| Nourkami-Tutdibi et al. | 2021 | EPICE | BPD, severe SNI (IVH ≥grade III or cystic PVL), severe ROP, and severe NEC |
| Pan, et al. | 2023 | China | ≥ grade III IVH or PVL, ≥ stage II NEC, severe BPD, or ≥ stage III or treatment-required ROP |
| Peng et al. | 2022 | China | SNI (IVH grade 3 or 4, or any grade of PVL determined by cranial imaging); NEC (Stage 2 or higher); moderate or severe BPD, and severe ROP |
| Serenius et al. | 2004 | Sweden | IVH grade 3–4 or PVL or ROP stage ≥3, BPD, i.e. supplemental oxygen at 36-week corrected GA and NEC |
| Simic et al. | 2014 | Sweden | severe ROP, severe IVH/cPVL, severe BPD, and NEC |
| Stoll et al. | 2010 | NICHD NRN | severe IVH (≥3), PVL, BPD, NEC, ≥ stage 3 ROP, or infection (early-onset sepsis, late-onset sepsis, or meningitis) |
| Stoll et al. | 2015 | NICHD NRN | NEC, infections (early-onset sepsis, late-onset sepsis, or meningitis), BPD, severe ICH, PVL, and ROP stage ≥ 3 |
| Wu et al. | 2021 | China | blood culture-positive sepsis, moderate to severe BPD, NEC ≥2 A, cystic PVL, IVH III/IV, severe ROP |
| Ye | 2024 | China | IVH grade 3-4/PVL, ROP grade 3-4, BPD, sepsis, or NEC≥2 A |
| Yeung et al. | 2024 | Canada, CNN | BPD, late-onset sepsis, NEC, SNI (IVH grade 3 or greater, or PVL or persistent parenchymal echogenicity, or severe ROP |
| Zayek et al. | 2011 | USA | WMI, NEC, BPD, or ROP stage 3 or more |
| Zhen et al. | 2023 | China | blood culture-positive sepsis, moderate to severe BPD, NEC≥2 A and necessitated surgery, IVH III/IV, severe ROP |
| Zhu et al. | 2021 | China | BPD, IVH (grade III-IV), WMI, NEC (stage II-III), sepsis, or severe ROP |

Abbreviations: BPD, bronchopulmonary dysplasia; CNN, Canadian Neonatal Network; CLD, chronic lung disease; cPVL, cystic periventricular leukomalacia; EPICE, Effective Perinatal Intensive Care in Europe for very preterm births; ICH, intracranial hemorrhage; IVH, intraventricular hemorrhage; NEC, necrotizing enterocolitis; NICHD, National Institute of Child Health and Human Development; NRN, Neonatal Research Network; PIVH, periventricular/ intraventricular hemorrhage; PVL, periventricular leukomalacia; ROP, retinopathy of prematurity; SNI, severe neurological injury; WMI, white matter injury.

**Additional file S10. Excluded full-text articles with reasons.**

| **References** | **Reason (number of articles)** |
| --- | --- |
| (1-18) | Abstracts or clinical trial registries without original data (18) |
| (19-45) | Conference proceedings, reviews, letters, note or editorials (27) |
| (46-50) | Non-English or Chinese (SWMM is not mentioned as well) (5) |
| (51-141) | Wrong outcomes (SWMM is not defined, or its definition does not meet the inclusion criteria; no specific number of SWMM events; SWMM were measured after hospital discharge) (92) |
| (142-199) | Wrong population (The study population does not consist of VPTs less than 32 weeks of gestation, or it is mixed with individuals with a GA greater than 32 weeks) (58) |
| (200, 201) | Duplicate publication (2) |
| (202) | Sample size <100 (1) |

**References**

1. Zeballos Sarrato S, Cuevas Del Campo L, Rodríguez Rojo M, Sánchez-Cabezudo Moreno C, Arriaga Redondo M, Sanchez Luna M. Changes in perinatal risk factors and outcomes of preterm infants <32 weeks gestational age in two different periods. Journal of Perinatal Medicine. (2015) 43. doi:10.1515/jpm-2015-2003

2. Tanner LD, Chauhan SP, Chen HY, Sibai BM. Racial and ethnic disparities in neonatal morbidity during the late preterm period. American Journal of Obstetrics and Gynecology. (2019) 220(1):S204-S5. doi:10.1016/j.ajog.2018.11.310

3. Pillai R, Tincello D, Potdar N. Threatened Miscarriage and increase in Perinatal Morbidity. Human Reproduction. (2021) 36(SUPPL 1):i316. doi:10.1093/humrep/deab130.396

4. Papadopoulou M, Agrogianni X, Biskini P, Gantseva S, Galanou S, Theodoraki M, et al. Neonatal mortality rate: A 14 year retrospective analysis. Journal of Perinatal Medicine. (2015) 43. doi:10.1515/jpm-2015-2003

5. Ozcan B, Kavurt S, Aydemir O, Gencturk Z, Bas AY, Demirel N. Snappe-II and risk of neonatal morbidities in very low birth weight preterm infants. Intensive Care Medicine. (2013) 39:S69-S70. doi:10.1007/s00134-013-2950-8

6. Muñoz-Garcia M, Millán-Miralles L, Santiago-Gutiérrez C, Martínez-Padilla MC, De La Cruz-Moreno J. Impact of neonatal morbidities on clinical outcome and predictors of mortality in preterm infants with low birth weigh. Archives of Disease in Childhood. (2014) 99:A462-A3. doi:10.1136/archdischild-2014-307384.1280

7. Marcussen JR, Birla S, Cheon T, Benedetto-Anzai MT, Sawai M, Anzai Y. Risk Factors for Preterm Birth: Analysis of U.S. Natality Data. Reproductive Sciences. (2023) 30:216A-7A. doi:10.1007/s43032-023-01211-1

8. Manea AM, Boia M, Iacob D, Dima M. Morbidity and mortality in extremely low birth weight newborn. Journal of Maternal-Fetal and Neonatal Medicine. (2014) 27:399-400. doi:10.3109/14767058.2014.924236

9. Herrera C, Silver RM, Major H, Varner MW, Clark EAS. Triple I criteria and adverse neonatal and childhood outcomes after early preterm birth. American Journal of Obstetrics and Gynecology. (2017) 216(1):S250.

10. Francis M, Chowdhury O, Young A. Maintaining excellence during change: implementing optimal cord management without compromising other perinatal outcomes. Archives of Disease in Childhood. (2024) 109:A131-A2. doi:10.1136/archdischild-2024-rcpch.193

11. Forde B, DeFranco E. Impact of prior cesarean delivery on early term delivery and neonatal morbidity. American Journal of Obstetrics and Gynecology. (2020) 222(1):S249-S50. doi:10.1016/j.ajog.2019.11.392

12. El Bakkali L. Spontaneous preterm delivery versus medically indicated preterm delivery-is there a difference in neonatal outcome? BJOG: An International Journal of Obstetrics and Gynaecology. (2016) 123:97. doi:10.1111/14710528.13988

13. de Andrade Ramos BR, Biazotto BM, Brisot HM, Joaquim RN, Góes G, Rodrigues I, et al. Implementation of a treatment bundle for risk factors for spontaneous preterm birth - Preliminary results. Placenta. (2024) 153:e36. doi:10.1016/j.placenta.2024.05.114

14. Dannecker C, Gallwas J, Hübener C, Rottmann M, Mahner S, Wetzka S. Preterm birth and conization prior to pregnancy: An analysis of the national inpatient quality survey data in Germany; 2009-2014. International Journal of Gynecological Cancer. (2017) 27:67. doi:10.1097/01.IGC.0000527296.86225.87

15. Corsello G, Giuffrè M. Post natal care and follow-up. Twin Research and Human Genetics. (2012) 15(2):184.

16. Bonnet C, Draper ES, Maier RF, Cuttini M, Herich L, Johnson SJ, et al. Contribution of severe neonatal morbidity to neurodevelopment at 2 years of age among very preterm infants: a mediation analysis. J Epidemiol Community Health. (2019) 73(Suppl 1):A63. doi:10.1136/jech-2019-SSMabstracts.133

17. Bental Y, Reichman B, Shiff Y, Weisbrod M, Boyko V, Lerner-Geva L, et al. Impact of maternal diabetes mellitus on mortality and morbidity of preterm infants (24-33 weeks' gestation). Pediatrics. (2011) 128(4):e848-55. doi:10.1542/peds.2010-3443

18. Ajibade F, Okoye O, Agwu S. Pregnancy outcome and maternal characteristic of patents with low PAPP-A. European Journal of Obstetrics Gynecology and Reproductive Biology. (2019) 234:e103. doi:10.1016/j.ejogrb.2018.08.386

19. Zeitlin J, Egorova NN, Janevic T, Hebert PL, Lebreton E, Balbierz A, et al. The impact of severe maternal morbidity on very preterm infant outcomes. Obstetrical and Gynecological Survey. (2020) 75(5):281-2. doi:10.1097/01.ogx.0000666236.10837.b5

20. Wen SW, Smith G, Yang Q, Walker M. Epidemiology of preterm birth and neonatal outcome. Seminars in Fetal and Neonatal Medicine. (2004) 9(6):429-35. doi:10.1016/j.siny.2004.04.002

21. Vidaeff AC, Ramin SM. From concept to practice: The recent history of preterm delivery prevention. Part II: Subclinical infection and hormonal effects. AMERICAN JOURNAL OF PERINATOLOGY. (2006) 23(2):75-84. doi:10.1055/s-2006-931803

22. Vanmathi SM, Monitha Star M, Venkateswaramurthy N, Sambath Kumar R. Preterm birth facts: A review. Research Journal of Pharmacy and Technology. (2019) 12(3):1383-90. doi:10.5958/0974-360X.2019.00231.2

23. Suresh P, Tagare A, Kadam S, Pandit AN. Neonatal sepsis and long term outcome. Journal of Neonatology. (2009) 23(1):64-7. doi:10.1177/0973217920090111

24. Song IG. Neurodevelopmental outcomes of preterm infants. Clinical and Experimental Pediatrics. (2023) 66(7):281-7. doi:10.3345/cep.2022.00822

25. Simeoni U, Haumont D. Coping with the dilemmas of extremely preterm birth: Outcome or ethics? Current Pediatric Reviews. (2013) 9(1):36-9. doi:10.2174/1573396311309010008

26. Shankaran S. Childhood neurodevelopmental outcome following extremely preterm birth. The Lancet Child and Adolescent Health. (2018) 2(12):843-4. doi:10.1016/S2352-4642(18)30308-0

27. Refuerzo JS. Impact of multiple births on late and moderate prematurity. Seminars in Fetal and Neonatal Medicine. (2012) 17(3):143-5. doi:10.1016/j.siny.2012.01.012

28. Pai VV, Kan P, Bennett M, Carmichael SL, Lee HC, Hintz SR, editors. Improved Referral of Very Low Birthweight Infants to High-Risk Infant Follow-Up in California. Journal of Pediatrics; 2020.

29. Norman M. Progress, Problems, and Prospects in the Intensive Care of Extremely Preterm Infants. JAMA. (2022) 327(3):225-6. doi:10.1001/jama.2021.22717

30. McCormick MC, Litt JS. The outcomes of very preterm infants: Is it time to ask different questions? Pediatrics. (2017) 139(1). doi:10.1542/peds.2016-1694

31. Kumar VHS. Special Issue: Prematurity, Preterm-Born Adults, and Long-Term Effects on Children and Adults. Children. (2023) 10(6). doi:10.3390/children10060989

32. Kramer MR, Hogue CJ, Dunlop AL, Menon R. Preconceptional stress and racial disparities in preterm birth: an overview. Acta Obstet Gynecol Scand. (2011) 90(12):1307-16. doi:10.1111/j.1600-0412.2011.01136.x

33. Janvier A, Shah PS. The premature lottery in the Canadian grey zones. Current Pediatric Reviews. (2013) 9(1):25-31. doi:10.2174/157339613805289541

34. Janevic T, Zeitlin J, Auger N, Egorova NN, Hebert P, Balbierz A, et al. Association of Race/Ethnicity with Very Preterm Neonatal Morbidities. Obstetrical and Gynecological Survey. (2019) 74(4):198-9. doi:10.1097/OGX.0000000000000667

35. Groenendaal F, Uiterwaal C. Long term follow-up of extremely preterm neonates. BMJ (Online). (2012) 345(7886). doi:10.1136/bmj.e8252

36. Gouyon JB, Iacobelli S, Ferdynus C, Bonsante F. Neonatal problems of late and moderate preterm infants. Seminars in Fetal and Neonatal Medicine. (2012) 17(3):146-52. doi:10.1016/j.siny.2012.01.015

37. Farooqi A, Serenius F. Outcome after preterm birth. Annales Nestle. (2005) 63(2):39-56.

38. Fanaroff AA, Fanaroff JM. Advocacy in Neonatology. American Journal of Perinatology. (2019) 36:S9-S12. doi:10.1055/s-0039-1691776

39. Engle WA. Morbidity and mortality in late preterm and early term newborns: A continuum. Clinics in Perinatology. (2011) 38(3):493-516. doi:10.1016/j.clp.2011.06.009

40. Drife J. Mode of delivery in the early preterm infant (<28 weeks). BJOG. (2006) 113(SUPPL. 3):81-5. doi:10.1111/j.1471-0528.2006.01129.x

41. Collin MF, Halsey CL, Anderson CL, editors. Emerging developmental sequelae in the 'normal' extremely low birth weight infant. Pediatrics; 1991.

42. Brown RN. Resolved threatened preterm labour: an opportunity for reducing future prematurity? BJOG: An International Journal of Obstetrics and Gynaecology. (2019) 126(7):906. doi:10.1111/1471-0528.15660

43. Blencowe H, Cousens S, Chou D, Oestergaard M, Say L, Moller AB, et al. Born Too Soon: The global epidemiology of 15 million preterm births. Reproductive Health. (2013) 10(SUPPL. 1). doi:10.1186/1742-4755-10-S1-S2

44. Ancel PY. Preterm labor: Pathophysiology, risk factors and outcomes. Journal de Gynecologie Obstetrique et Biologie de la Reproduction. (2002) 31(7 SUPPL.):5S10-5S21.

45. Al Nemri A. Immediate and neuro-developmental outcome of extremely low birth weight (ELBW) below 750 grams: 5 Years experience in King Khalid University Hospital, Riyadh. Current Pediatric Research. (2004) 8(1-2):15-8.

46. Trotter A. Quality of Care for Very Low Birth Weight Infants in Germany - Evaluation of Publicly Available Data from 2014 to 2018. Zeitschrift fur Geburtshilfe und Neonatologie. (2021) 225(1):74-9. doi:10.1055/a-1350-3953

47. Salas N R, Sanhueza U L, Maggi C L. Risk factors and clinical evolution in premature infants less than 1 000 g of weight. Revista Chilena de Pediatria. (2006) 77(6):577-88.

48. Ombelet W. Multiple birth prevention in assisted reproduction. Tijdschrift voor Fertiliteitsonderzoek. (2005) 19(4):78-86.

49. D'Angiolella ML, Battista L, Gambardella V, D'Ambrosio M, Vitelli A, Sodano A, et al. Risk factors and neonatal morbidity in prematurity: Sperimental observations. Giornale Italiano di Ostetricia e Ginecologia. (2003) 25(4):143-7.

50. Ben Hamida Nouaili E, Ben Ammar H, Zouari B, Chaouachi S, Marrakchi Z. Neonatal mortality and morbidity in newborn newborns of single mothers. Tunisie Medicale. (2008) 86(1):9-11.

51. Abdel-Latif ME, Bajuk B, Oei J, Lui K. Mortality and morbidities among very premature infants admitted after hours in an Australian neonatal intensive care unit network. Pediatrics. (2006) 117(5):1632-9. doi:10.1542/peds.2005-1421

52. Aliyu MH, Salihu HM, Keith LG, Ehiri JE, Islam MA, Jolly PE. High parity and fetal morbidity outcomes. Obstetrics and Gynecology. (2005) 105(5 I):1045-51. doi:10.1097/01.AOG.0000157444.74674.75

53. Al-Mouqdad MM, Alshaikh B, Sumaily HH, Almotiri AA, Alodhaidan NA, AlMahmoud L, et al. Impact of Refeeding Syndrome on the Short-Term Clinical Outcomes of Very-Premature Infants. Nutrients. (2024) 16(20). doi:10.3390/nu16203445

54. Audu LI, Otuneye AT, Mairami AB, Mukhtar-Yola M, Mshelia LJ. Determination of neonatal case-specific fatality rates in a tertiary health institution in North Central Nigeria. BMC Pediatrics. (2021) 21(1). doi:10.1186/s12887-021-02778-x

55. Azria E, Kayem G, Langer B, Marchand-Martin L, Marret S, Fresson J, et al. Neonatal mortality and long-term outcome of infants born between 27 and 32 weeks of gestational age in breech presentation: The EPIPAGE cohort study. PLoS ONE. (2016) 11(1). doi:10.1371/journal.pone.0145768

56. Bacak SJ, Baptiste-Roberts K, Amon E, Ireland B, Leet T. Risk factors for neonatat mortatity among extremety-tow-birth-weight infants. AMERICAN JOURNAL OF OBSTETRICS AND GYNECOLOGY. (2005) 192(3):862-7. doi:10.1016/j.ajog.2004.07.029

57. Bader D, Kugelman A, Boyko V, Levitzki O, Lerner-Geva L, Riskin A, et al. Risk Factors and Estimation Tool for Death Among Extremely Premature Infants: A National Study. PEDIATRICS. (2010) 125(4):696-703. doi:10.1542/peds.2009-1607

58. Bajwa NM, Berner M, Worley S, Pfister RE, Zeilinger G, Bührer C, et al. Population-based age stratified morbidities of premature infants in Switzerland. Swiss Medical Weekly. (2011) 141(JUNE). doi:10.4414/smw.2011.13212

59. Bertino E, Coscia A, Boni L, Rossi C, Martano C, Giuliani F, et al. Weight growth velocity of very low birth weight infants: role of gender, gestational age and major morbidities. Early Human Development. (2009) 85(6):339-47. doi:10.1016/j.earlhumdev.2008.12.014

60. Bezzine A, Chebbi I, Hamida EB, Marrakchi Z. In-hospital mortality of very preterm infants in a Tunisian neonatal intensive care unit: Prevalence and risk factors. Tunisie Medicale. (2018) 96(12):884-7.

61. Bolisetty S, Legge N, Bajuk B, Lui K. Preterm infant outcomes in New South Wales and the Australian Capital Territory. J. Paediatr. Child Health. (2015) 51(7):713-21. doi:10.1111/jpc.12848

62. Bottoms SF, Paul RH, Mercer BM, MacPherson CA, Caritis SN, Moawad AH, et al. Obstetric determinants of neonatal survival: Antenatal predictors of neonatal survival and morbidity in extremely low birth weight infants. American Journal of Obstetrics and Gynecology. (1999) 180(3 I):665-9. doi:10.1016/S0002-9378(99)70270-X

63. Brettell R, Yeh PS, Impey LWM. Examination of the association between male gender and preterm delivery. Eur J Obstet Gynecol Reprod Biol. (2008) 141(2):123-6. doi:10.1016/j.ejogrb.2008.07.030

64. Byram SK, Penumaka C, Bai VK, Mandala H, Shanti P. A prospective study of maternal risk factors in relation to preterm babies and their immediate outcome in a tertiary care hospital, Guntur. International Journal of Academic Medicine and Pharmacy. (2024) 6(4):430-7. doi:10.47009/jamp.2024.6.4.88

65. Chan K, Ohlsson A, Synnes A, Lee DSC, Chien LY, Lee SK, et al. Survival, morbidity, and resource use of infants of 25 weeks' gestational age or less. Am J Obstet Gynecol. (2001) 185(1):220-6. doi:10.1067/mob.2001.115280

66. Charkaluk ML, Truffert P, Fily A, Ancel PY, Pierrat V, Epipage Study G. Neurodevelopment of children born very preterm and free of severe disabilities: the Nord-Pas de Calais Epipage cohort study. Acta Paediatr. (2010) 99(5):684-9. doi:10.1111/j.1651-2227.2010.01695.x

67. Chee YY, Wong MSC, Wong RMS, Wong KY. Neonatal outcomes of preterm or very-low-birth-weight infants over a decade from Queen Mary Hospital, Hong Kong: comparison with the Vermont Oxford Network. Hong Kong Med J. (2017) 23(4):381-6. doi:10.12809/hkmj166064

68. Chen F, Bajwa NM, Rimensberger PC, Posfay-Barbe KM, Pfister RE, Swiss Neonatal N. Thirteen-year mortality and morbidity in preterm infants in Switzerland. Arch Dis Child Fetal Neonatal Ed. (2016) 101(5):F377-F83. doi:10.1136/archdischild-2015-308579

69. Choi EK, Kim HJ, Je BK, Choi BM, Kim SD. Morbidity and Mortality Trends in Preterm Infants of <32 Weeks Gestational Age with Severe Intraventricular Hemorrhage : A 14-Year Single-Center Retrospective Study. J. Korean Neurosurg. Soc. (2023) 66(3):316-23. doi:10.3340/jkns.2022.0264

70. De Almeida MFB, Guinsburg R, Martinez FE, Procianoy RS, Leone CR, Marba STM, et al. Perinatal factors associated with early deaths of preterm infants born in Brazilian Network on Neonatal Research centers. Jornal de Pediatria. (2008) 84(4):300-7. doi:10.2223/JPED.1787

71. De Kleine MJK, Den Ouden AL, Kollée LAA, Ilsen A, Van Wassenaer AG, Brand R, et al. Lower mortality but higher neonatal morbidity over a decade in very preterm infants. Paediatric and Perinatal Epidemiology. (2007) 21(1):15-25. doi:10.1111/j.1365-3016.2007.00780.x

72. EXPRESS Group. Incidence of and risk factors for neonatal morbidity after active perinatal care: extremely preterm infants study in Sweden (EXPRESS). Acta Paediatr. (2010) 99(7):978-92. doi:10.1111/j.1651-2227.2010.01846.x

73. Gamini BS, Charki S, Patil MM, Bidri SR, Patil N, Kalyanshettar SS, et al. Late preterm births and associated neonatal outcomes in a tertiary care center: A prospective observational study. Current Pediatric Research. (2021) 25(7):677-84.

74. García-Muñoz Rodrigo F, Fabres JG, Zozaya Nieto C, San Feliciano L, Figueras-Aloy J, Saenz de Pipaon M, et al. Survival and Survival without Major Morbidity Seem to Be Consistently Better throughout Gestational Age in 24- to 30-Week Gestational Age Very-Low-Birth-Weight Female Infants Compared to Males. Neonatology. (2022) 119(5):585-93.

75. Garciá-Muñoz Rodrigo F, Fabres J, Tapia JL, D'Apremont I, San Feliciano L, Zozaya Nieto C, et al. Factors Associated with Survival and Survival without Major Morbidity in Very Preterm Infants in Two Neonatal Networks: SEN1500 and NEOCOSUR. Neonatology. (2021) 118(3):289-96. doi:10.1159/000513079

76. Gladstone M, White S, Kafulafula G, Neilson JP, van den Broek N. Post-neonatal mortality, morbidity, and developmental outcome after ultrasound-dated preterm birth in rural malawi: A community-based cohort study. PLoS Medicine. (2011) 8(11). doi:10.1371/journal.pmed.1001121

77. Godeluck A, Gérardin P, Lenclume V, Mussard C, Robillard PY, Sampériz S, et al. Mortality and severe morbidity of very preterm infants: Comparison of two French cohort studies. BMC Pediatrics. (2019) 19(1). doi:10.1186/s12887-019-1700-7

78. Group S-NNNC, Yonghui Y. Etiology of preterm birth and in-hospital adverse outcome of very preterm infants: a multicenter prospective observational cohort study. Chinese Journal of Perinatal Medicine. (2023) 26(5):357-65. doi:10.3760/cma.j.cn113903-20221223-01047

79. Guimaraes P, Joao A, Costa M, Manuela F. Prematurity morbidity: First year of life. Nascer e Crescer. (1998) 7(1):26-8.

80. Hamilton EF, Dyachenko A, Ciampi A, Maurel K, Warrick PA, Garite TJ. Estimating risk of severe neonatal morbidity in preterm births under 32 weeks of gestation. Journal of Maternal-Fetal and Neonatal Medicine. (2020) 33(1):73-80. doi:10.1080/14767058.2018.1487395

81. Härkin P, Marttila R, Pokka T, Saarela T, Hallman M. Survival analysis of a cohort of extremely preterm infants born in Finland during 2005–2013. Journal of Maternal-Fetal and Neonatal Medicine. (2021) 34(15):2506-12. doi:10.1080/14767058.2019.1668925

82. Hays SP, Smith EOB, Sunehag AL. Hyperglycemia is a risk factor for early death and morbidity in extremely low birth-weight infants. Pediatrics. (2006) 118(5):1811-8. doi:10.1542/peds.2006-0628

83. Hempenius MA, Verhagen EA, Tanis JC, Einspieler C, Bos AF. Early neonatal morbidities and neurological functioning of preterm infants 2 weeks after birth. Journal of Perinatology. (2018) 38(11):1518-25. doi:10.1038/s41372-018-0211-y

84. Heuckendorff S, Christensen LF, Fonager K, Overgaard C. Risk of adverse perinatal outcomes in infants born to mothers with mental health conditions. Acta Obstetricia et Gynecologica Scandinavica. (2021) 100(11):2019-28. doi:10.1111/aogs.14241

85. Huang HB, Hicks M, Zhang QS, Watt MJ, Lin F, Wan XQ, et al. The differential associative relationship between early risk factors, neonatal morbidities and early neurodevelopmental outcome in preterm infants <29 weeks' gestation. Early Human Development. (2023) 186. doi:10.1016/j.earlhumdev.2023.105859

86. Ito M, Tamura M, Namba F. Role of sex in morbidity and mortality of very premature neonates. Pediatrics international : official journal of the Japan Pediatric Society. (2017) 59(8):898-905. doi:10.1111/ped.13320

87. Janevic T, Zeitlin J, Auger N, Egorova NN, Hebert P, Balbierz A, et al. Association of Race/Ethnicity with Very Preterm Neonatal Morbidities. JAMA Pediatrics. (2018) 172(11):1061-9. doi:10.1001/jamapediatrics.2018.2029

88. Ji X, Wu C, Chen M, Wu L, Li T, Miao Z, et al. Analysis of risk factors related to extremely and very preterm birth: a retrospective study. BMC Pregnancy Childbirth. (2022) 22(1). doi:10.1186/s12884-022-05119-7

89. Kacperczyk-Bartnik J, Bartnik P, Teliga-Czajkowska J, Malinowska-Polubiec A, Dobrowolska-Redo A, Romejko-Wolniewicz E, et al. Risk factors associated with neonatal infectious and respiratory morbidity following preterm premature rupture of membranes. Ginekol. Pol. (2022) 93(8):629-36. doi:10.5603/GP.a2022.0066

90. Kaempf J, Morris M, Steffen E, Wang L, Dunn M. Continued improvement in morbidity reduction in extremely premature infants. Archives of Disease in Childhood: Fetal and Neonatal Edition. (2021) 106(3):F265-F70. doi:10.1136/archdischild-2020-319961

91. Kalk P, Guthmann F, Krause K, Relle K, Godes M, Gossing G, et al. Impact of maternal body mass index on neonatal outcome. European journal of medical research. (2009) 14(5):216-22.

92. Khanum A, Lavereen S, Moniruzzaman, Romana. Risk factors and outcome of preterm labour in Tertiary health centre. Bangladesh Journal of Obstetrics and Gynecology. (2020) 32(2):90-3. doi:10.3329/BJOG.V32I2.48279

93. Kono Y, Yonemoto N, Nakanishi H, Hosono S, Hirano S, Kusuda S, et al. A Retrospective Cohort Study on Mortality and Neurodevelopmental Outcomes of Preterm Very Low Birth Weight Infants Born to Mothers with Hypertensive Disorders of Pregnancy. American Journal of Perinatology. (2022) 39(13):1465-77. doi:10.1055/s-0041-1722874

94. Kugelman A, Bader D, Lerner-Geva L, Boyko V, Levitzki O, Riskin A, et al. Poor outcomes at discharge among extremely premature infants: A national population-based study. Archives of Pediatrics and Adolescent Medicine. (2012) 166(6):543-50. doi:10.1001/archpediatrics.2011.891

95. Layden AJZ. Investigating Placental Inflammation as a Mediator of Maternal Obesity and Risk of Preterm Birth2022 2022.

96. Leonard CH, Clyman RI, Piecuch RE, Juster RP, Ballard RA, Behle MB. Effect of medical and social risk factors on outcome of prematurity and very low birth weight. The Journal of pediatrics. (1990) 116(4):620-6. doi:10.1016/S0022-3476(05)81616-6

97. Li ZW, Gu MQ, Xu XZ, Tang LF, Xu XY, Yang JH, et al. Analysis of Clinical Data of 101 Extremely Premature Infants in a Tertiary Grade A Hospital. Journal of Kunming Medical University. (2022) 43(06):85-91.

98. Lundeby KM, Heen E, Mosa M, Abdi A, Størdal K. Neonatal morbidity and mortality in Hargeisa, Somaliland: An observational, hospital based study. Pan African Medical Journal. (2020) 37:1-15. doi:10.11604/pamj.2020.37.3.24741

99. Mansouri HA. Perinatal factors and neonatal outcome of very low birth weight and extremely premature babies at KAUH. Bahrain Medical Bulletin. (2001) 23(2):66-71.

100. Manuck TA, Rice MM, Bailit JL, Grobman WA, Reddy UM, Wapner RJ, et al. Preterm neonatal morbidity and mortality by gestational age: a contemporary cohort. Am J Obstet Gyneco. (2016) 215(1). doi:10.1016/j.ajog.2016.01.004

101. Misra S, Yadav AK. A study of adverse outcome in early and moderate preterms. Indian Journal of Public Health Research and Development. (2016) 7(1):17-22. doi:10.5958/0976-5506.2016.00005.X

102. Miyazaki K, Furuhashi M, Ishikawa K, Tamakoshi K, Hayashi K, Kai A, et al. Impact of chorioamnionitis on short- and long-term outcomes in very low birth weight preterm infants: the Neonatal Research Network Japan. J Matern Fetal Neonatal Med. (2016) 29(2):331-7. doi:10.3109/14767058.2014.1000852

103. Mukhtar-Yola M, Iliyasu Z. A review of neonatal morbidity and mortality in Aminu Kano Teaching Hospital, northern Nigeria. Tropical Doctor. (2007) 37(3):130-2. doi:10.1258/004947507781524683

104. Nabwera HM, Wang D, Tongo OO, Andang’o PEA, Abdulkadir I, Ezeaka CV, et al. Burden of disease and risk factors for mortality amongst hospitalized newborns in Nigeria and Kenya. PLoS ONE. (2021) 16(1 January). doi:10.1371/journal.pone.0244109

105. Nkeeto B, Yawe BL, Matovu F. Determinants associated with preterm births in Uganda: A cross-sectional study. 2022.

106. Ovaskainen K, Ojala R, Gissler M, Luukkaala T, Tammela O. Out-of-hospital deliveries have risen involving greater neonatal morbidity: Risk factors in out-of-hospital deliveries in one University Hospital region in Finland. Acta Paediatrica, International Journal of Paediatrics. (2015) 104(12):1248-52. doi:10.1111/apa.13117

107. Özcan B, Kavurt AS, Aydemir Ö, Gençtürk Z, Baş AY, Demirel N. SNAPPE-II and risk of neonatal morbidities in very low birth weight preterm infants. Turkish Journal of Pediatrics. (2017) 59(2):105-12. doi:10.24953/turkjped.2017.02.001

108. Pangratz-Fuehrer S, Genzel-Boroviczény O, Bodensohn WE, Eisenburger R, Scharpenack J, Geyer PE, et al. Cohort profile: The MUNICH Preterm and Term Clinical study (MUNICH-PreTCl), a neonatal birth cohort with focus on prenatal and postnatal determinants of infant and childhood morbidity. BMJ Open. (2021) 11(6). doi:10.1136/bmjopen-2021-050652

109. Paniagua U, Lester BM, Marsit CJ, Camerota M, Carter BS, Check JF, et al. Epigenetic age acceleration, neonatal morbidities, and neurobehavioral profiles in infants born very preterm. Epigenetics. (2023) 18(1). doi:10.1080/15592294.2023.2280738

110. Patra K, Greene MM, Patel AL, Meier P. Maternal Education Level Predicts Cognitive, Language, and Motor Outcome in Preterm Infants in the Second Year of Life. American Journal of Perinatology. (2016) 33(8):738-44. doi:10.1055/s-0036-1572532

111. Paz-Zulueta M, Llorca J, Sarabia-Lavín R, Bolumar F, Rioja L, Delgado A, et al. The role of prenatal care and social risk factors in the relationship between immigrant status and neonatal morbidity: A Retrospective cohort study. PLoS ONE. (2015) 10(3). doi:10.1371/journal.pone.0120765

112. Peacock JL, Marston L, Marlow N, Calvert SA, Greenough A. Neonatal and infant outcome in boys and girls born very prematurely. Pediatr Res. (2012) 71(3):305-10. doi:10.1038/pr.2011.50

113. Petrova A, Demissie K, Rhoads GG, Smulian JC, Marcella S, Ananth CV. Association of maternal fever during labor with neonatal and infant morbidity and mortality. Obstetrics and Gynecology. (2001) 98(1):20-7. doi:10.1016/S0029-7844(01)01361-8

114. Raisanen S, Gissler M, Saari J, Kramer M, Heinonen S. Contribution of Risk Factors to Extremely, Very and Moderately Preterm Births - Register-Based Analysis of 1,390,742 Singleton Births. PLOS ONE. (2013) 8(4). doi:10.1371/journal.pone.0060660

115. Ruiz E, Piamonte DE, Gómez DT, Díaz LA, Pérez LA. Incidence of metabolic bone disease in neonates under 32 gestational weeks at the Hospital Universitario de Santander in Colombia. Biomedica : revista del Instituto Nacional de Salud. (2024) 44(1):35-44. doi:10.7705/biomedica.6926

116. Sadeghzadeh M, Khoshnevisasl P, Parvaneh M, Mousavinasab N. Early and Late Outcome of Premature Newborns with History of Neonatal Intensive Care Units Admission at 6 Years Old in Zanjan, Northwestern Iran. Iranian journal of child neurology. (2016) 10(2):67-73.

117. Saldir M, Sarici SU, Bakar EE, Özcan O. Neurodevelopmental status of preterm newborns at infancy, born at a Tertiary Care Center in Turkey. American Journal of Perinatology. (2010) 27(2):121-8. doi:10.1055/s-0029-1224863

118. Salihu HM, Lynch O, Alio AP, Mbah AK, Kornosky JL, Marty PJ. Extreme maternal underweight and feto-infant morbidity outcomes: A population-based study. Journal of Maternal-Fetal and Neonatal Medicine. (2009) 22(5):428-34. doi:10.1080/14767050802385764

119. Schenone MH, Aguin E, Li Y, Lee C, Kruger M, Bahado-singh RO. Prenatal prediction of neonatal survival at the borderline viability. The Journal of Maternal-Fetal & Neonatal Medicine. (2010) 23(12):1413-8. doi:10.3109/14767058.2010.481318

120. Shim SY, Cho SJ, Kong KA, Park EA. Gestational age-specific sex difference in mortality and morbidities of preterm infants: A nationwide study. Sci. Rep. (2017) 7(1):6161. doi:10.1038/s41598-017-06490-8

121. Sotiriadis A, Eleftheriades M, Papadopoulos V, Sarafidis K, Pervanidou P, Assimakopoulos E. Divergence of estimated fetal weight and birth weight in singleton fetuses. Journal of Maternal-Fetal and Neonatal Medicine. (2018) 31(6):761-9. doi:10.1080/14767058.2017.1297409

122. Srinivasjois R, Nembhard W, Wong K, Bourke J, Pereira G, Leonard H. Risk of Mortality into Adulthood According to Gestational Age at Birth. Journal of Pediatrics. (2017) 190:185-91.e1. doi:10.1016/j.jpeds.2017.07.051

123. Su Z, Liang S, Huang X, Wu H, Wei J, Jia C, et al. Effects of gender on clinical outcomes in extremely low birth weight infants and analysis of risk factors of mortality. Chinese Journal of Neonatology. (2022) 37(02):138-42. doi:10.3760/cma.j.issn.2096-2932.2022.02.009

124. Suciu LM, Puscasiu L, Szabo B, Cucerea M, Ognean ML, Oprea I, et al. Mortality and morbidity of very preterm infants in Romania: how are we doing? Pediatrics international : official journal of the Japan Pediatric Society. (2014) 56(2):200-6. doi:10.1111/ped.12219

125. Sullivan BA, Doshi A, Chernyavskiy P, Husain A, Binai A, Sahni R, et al. Neighborhood Deprivation and Association with Neonatal Intensive Care Unit Mortality and Morbidity for Extremely Premature Infants. JAMA Network Open. (2023) 6(5):E2311761. doi:10.1001/jamanetworkopen.2023.11761

126. Tette EMA, Nartey ET, Nuertey BD, Azusong EA, Akaateba D, Yirifere J, et al. The pattern of neonatal admissions and mortality at a regional and district hospital in the Upper West Region of Ghana; a cross sectional study. PLoS ONE. (2020) 15(5). doi:10.1371/journal.pone.0232406

127. Thatrimontrichai A, Phatigomet M, Maneenil G, Dissaneevate S, Janjindamai W. Risk Factors for Mortality or Major Morbidities of Very Preterm Infants: A Study from Thailand. Am. J. Perinatol. (2024) 41(10):1379-87. doi:10.1055/a-2016-7568

128. Tomo CK, Balogun OO, Davidson J, Guinsburg R, de Almeida MFB, de Andrade Lopes JM, et al. Comparison of mortality and survival without major morbidities of very preterm infants with very low birth weight from Japan and Brazil. Revista Paulista de Pediatria. (2023) 41. doi:10.1590/1984-0462/2023/41/2021389

129. Townsel C, Hesson AM, Greco P, Kazzi NG, Treadwell MC. The Impact of Spontaneous versus Indicated Preterm Birth on Neonatal Outcomes among Extremely Premature Neonates. American Journal of Perinatology. (2024) 41:E2985-E9. doi:10.1055/a-2184-1374

130. Tran HT, Doyle LW, Lee KJ, Dang NM, Graham SM. Morbidity and mortality in hospitalised neonates in central Vietnam. Acta Paediatrica, International Journal of Paediatrics. (2015) 104(5):e200-e5. doi:10.1111/apa.12960

131. Vanhaesebrouck S, Zecic A, Goossens L, Keymeulen A, Garabedian L, De Meulemeester J, et al. Trends in neonatal morbidity and mortality for very low birthweight infants: a 20-year single-center experience. J Matern Fetal Neonatal Med. (2023) 36(2):2227311. doi:10.1080/14767058.2023.2227311

132. Verma R, Shibly S, Fang H, Pollack S. Do early postnatal body weight changes contribute to neonatal morbidities in the extremely low birth weight infants. Journal of Neonatal-Perinatal Medicine. (2015) 8(2):113-8. doi:10.3233/NPM-15814104

133. Villar J, Restrepo-Méndez MC, McGready R, Barros FC, Victora CG, Munim S, et al. Association between Preterm-Birth Phenotypes and Differential Morbidity, Growth, and Neurodevelopment at Age 2 Years: Results from the INTERBIO-21st Newborn Study. JAMA Pediatrics. (2021) 175(5):483-93. doi:10.1001/jamapediatrics.2020.6087

134. Vogel JP, Lee ACC, Souza JP. Maternal morbidity and preterm birth in 22 low- and middle-income countries: A secondary analysis of the WHO Global Survey dataset. BMC Pregnancy and Childbirth. (2014) 14(1). doi:10.1186/1471-2393-14-56

135. Yeo KT, Lee QY, Quek WS, Wang YA, Bolisetty S, Lui K. Trends in morbidity and mortality of extremely preterm multiple gestation newborns. Pediatrics. (2015) 136(2):263-71. doi:10.1542/peds.2014-4075

136. Zapata-Vázquez RE, Rodríguez-Carvajal LA, Sierra-Basto G, Alonzo-Vázquez FM, Echeverría-Eguíluz M. Discriminant function of perinatal risk that predicts early neonatal morbidity: Its validity and reliability. Archives of Medical Research. (2003) 34(3):214-21. doi:10.1016/S0188-4409(03)00029-8

137. Zeitlin J, Gwanfogbe CD, Delmas D, Pilkington H, Jarreau PH, Chabernaud JL, et al. Risk factors for not delivering in a level III unit before 32 weeks of gestation: Results from a population-based study in Paris and surrounding districts in 2003. Paediatric and Perinatal Epidemiology. (2008) 22(2):126-35. doi:10.1111/j.1365-3016.2007.00921.x

138. Zeitlin J, Manktelow BN, Piedvache A, Cuttini M, Boyle E, van Heijst A, et al. Use of evidence based practices to improve survival without severe morbidity for very preterm infants: results from the EPICE population based cohort. (2016) (1756-1833 (Electronic)).

139. Zhou WQ, Mei YB, Zhang XY, Li QP, Kong XY, Feng ZC. Neonatal outcomes of very preterm infants from a neonatal intensive care center. World Journal of Pediatrics. (2014) 10(1):53-8. doi:10.1007/s12519-013-0445-x

140. Aronsson E, Holzmann M, Bartocci M, Hulthén Varli I, Saltvedt S. Changes in perinatal management and outcomes of extremely preterm infants born below 26 weeks of gestation in a tertiary referral hospital in Sweden: Comparison between 2004–2007 and 2012–2016. Acta Obstetricia et Gynecologica Scandinavica. (2023) 102(6):716-27. doi:10.1111/aogs.14576

141. Boo NY, Chee SC, Neoh SH, Ang EB, Ang EL, Choo P, et al. Ten-year trend of care practices, morbidities and survival of very preterm neonates in the Malaysian National Neonatal Registry: a retrospective cohort study. BMJ Paediatr Open. (2021) 5(1):e001149. doi:10.1136/bmjpo-2021-001149

142. Aiken CEM, Fowden AL, Smith GCS. Antenatal glucocorticoids prior to cesarean delivery at term. JAMA Pediatrics. (2014) 168(6):507-8. doi:10.1001/jamapediatrics.2014.9

143. Ayari F, Sdiri Y, Cherifi E, Belhaj Ammar W, Chourou H, Kacem S, et al. Newborns of preeclamptic mothers: Morbidity and mortality in a level 3 maternity hospital. Current Pediatric Research. (2021) 25(12).

144. Baig R, Mutukulla R, Polanki R, Kalyani S. Morbidity and mortality pattern of late preterm neonates admitted in a tertiary care centre. International Journal of Academic Medicine and Pharmacy. (2022) 4(4):172-80. doi:10.47009/jamp.2022.4.4.35

145. Beeckman K, van De Putte S, Putman K, Louckx F. Predictive social factors in relation to preterm birth in a metropolitan region. Acta Obstet. Gynecol. Scand. (2009) 88(7):787-92. doi:10.1080/00016340902974007

146. Boghossian NS, Saha S, Bell EF, Brumbaugh JE, Shankaran S, Carlo WA, et al. Birth weight discordance in very low birth weight twins: mortality, morbidity, and neurodevelopment. J Perinatol. (2019) 39(9):1229-40. doi:10.1038/s41372-019-0427-5

147. Brown HK, Speechley KN, Macnab J, Natale R, Campbell MK. Neonatal morbidity associated with late preterm and early term birth: The roles of gestational age and biological determinants of preterm birth. International Journal of Epidemiology. (2014) 43(3):802-14. doi:10.1093/ije/dyt251

148. Chanvitan P, Ruangnapa K, Janjindamai W, Disaneevate S. Outcomes of very low birth weight infants in Songklanagarind Hospital. Journal of the Medical Association of Thailand = Chotmaihet thangphaet. (2010) 93(2):191-8.

149. Dahman HAB. Risk factors associated with preterm birth: a retrospective study in Mukalla Maternity and Childhood Hospital, Hadhramout Coast/Yemen. Sudanese journal of paediatrics. (2020) 20(2):99-110. doi:10.24911/SJP.106-1575722503

150. Debevec T, Burtscher J, Millet GP. Preterm birth: Potential risk factor for greater COVID-19 severity?. Respir Physiol Neurobiol. 2020;280:103484. doi:10.1016/j.resp.2020.103484

151. Fritz T, Källén K, Maršál K, Jacobsson B. Outcome of extremely preterm infants after iatrogenic or spontaneous birth. Acta Obstet. Gynecol. Scand. (2018) 97(11):1388-95. doi:10.1111/aogs.13387

152. Garcia-Munoz Rodrigo F, Galan Henriquez GM, Gomez Ospina C. Morbidity and Mortality Among Very-low-birth-weight Infants Born to Mothers with Clinical Chorioamnionitis. Pediatr Neonatol. (2014) 55(5):381-6. doi:10.1016/j.pedneo.2013.12.007

153. Germany L, Saurel-Cubizolles MJ, Ehlinger V, Napoletano A, Alberge C, Guyard-Boileau B, et al. Social context of preterm delivery in France in 2011 and impact on short-term health outcomes: the EPIPAGE 2 cohort study. Paediatr. Perinat. Epidemiol. (2015) 29(3):184-95. doi:10.1111/ppe.12189

154. Girsen AI, Mayo JA, Carmichael SL, Phibbs CS, Shachar BZ, Stevenson DK, et al. Women's prepregnancy underweight as a risk factor for preterm birth: a retrospective study. BJOG. (2016) 123(12):2001-7. doi:10.1111/1471-0528.14027

155. Govindaswami B, Nudelman M, Narasimhan SR, Huang A, Misra S, Urquidez G, et al. Eliminating Risk of Intubation in Very Preterm Infants with Noninvasive Cardiorespiratory Support in the Delivery Room and Neonatal Intensive Care Unit. Biomed Res Int. (2019) 2019:5984305. doi:10.1155/2019/5984305

156. Hellmeyer L, Herz K, Liedtke B, Wohlmuth P, Schmidt S, Hackeloeer BJ. The underestimation of immaturity in late preterm infants. Archives of Gynecology and Obstetrics. (2012) 286(3):619-26. doi:10.1007/s00404-012-2366-7

157. Hughes K, Sim S, Roman A, Michalak K, Kane S, Sheehan P. Outcomes and predictive tests from a dedicated specialist clinic for women at high risk of preterm labour: A ten year audit. Aust N Z J Obstet Gynaecol.. (2017) 57(4):405-11. doi:10.1111/ajo.12610

158. Jegatheesan P, Belogolovsky E, Nudelman M, Narasimhan SR, Huang A, Govindaswami B, et al. Longer Duration of Cord Clamping Improves Nicu Survival Without Major Morbidities in Very Preterm Infants. Children. (2024) 11(12). doi:10.3390/children11121546

159. Jeschke E, Biermann A, Guenster C, Boehler T, Heller G, Hummler HD, et al. Mortality and Major Morbidity of Very-Low-Birth-Weight Infants in Germany 2008-2012: A Report Based on Administrative Data. FRONTIERS IN PEDIATRICS. (2016) 4. doi:10.3389/fped.2016.00023

160. Kilpatrick SJ, Abreo A, Gould J, Greene N, Main EK. Confirmed severe maternal morbidity is associated with high rate of preterm delivery. Am. J. Obstet. Gynecol. (2016) 215(2):233.e1-7. doi:10.1016/j.ajog.2016.02.026

161. Kim CR, Vohr BR, Oh W. Effects of maternal hypertension in very-low-birth-weight infants. Archives of pediatrics & adolescent medicine. (1996) 150(7):686-91. doi:10.1001/archpedi.1996.02170320032005

162. Klinger G, Reichman B, Sirota L, Lusky A, Linder N. Risk factors for delayed discharge home in very-low-birthweight infants - A population-based study. Acta Paediatrica, International Journal of Paediatrics. (2005) 94(11):1674-9. doi:10.1080/08035250510046722

163. Laffan EE, McNamara PJ, Amaral J, Whyte H, L'Herault J, Temple M, et al. Review of interventional procedures in the very low birth-weight infant (<1.5 kg): Complications, lessons learned and current practice. Pediatric Radiology. (2009) 39(8):781-90. doi:10.1007/s00247-009-1267-9

164. Lawrence ER, Beyuo TK, Kobernik EK, Moyer CA, Oppong SA. A comparative analysis of neonatal outcomes in pregnancies complicated by preeclampsia and eclampsia in Ghana. AJOG global reports. (2022) 2(3). doi:10.1016/j.xagr.2022.100061

165. Lee HC, Liu J, Profit J, Hintz SR, Gould JB. Survival Without Major Morbidity Among Very Low Birth Weight Infants in California. Pediatrics. (2020) 146(1):e20193865.

166. Lee SM, Lee MH, Chang YS. The Clinical Risk Index for Babies II for Prediction of Time-Dependent Mortality and Short-Term Morbidities in Very Low Birth Weight Infants. Neonatology. (2019) 116(3):244-51. doi:10.1159/000500270

167. Lodha A, Zhu Q, Lee SK, Shah PS, Andrews W, Barrington K, et al. Neonatal outcomes of preterm infants in breech presentation according to mode of birth in Canadian NICUs. Postgraduate Medical Journal. (2011) 87(1025):175-9. doi:10.1136/pgmj.2010.107532

168. Lorthe E, Sentilhes L, Quere M, Lebeaux C, Winer N, Torchin H, et al. Planned delivery route of preterm breech singletons, and neonatal and 2-year outcomes: a population-based cohort study. BJOG : an international journal of obstetrics and gynaecology. (2019) 126(1):73-82. doi:10.1111/1471-0528.15466

169. Mengistu TS, Schreiber V, Flatley C, Fox J, Kumar S. Factors associated with increased risk of early severe neonatal morbidity in late preterm and early term infants. Journal of Clinical Medicine. (2021) 10(6). doi:10.3390/jcm10061319

170. Minor KC, Bianco K, Sie L, Druzin ML, Lee HC, Leonard SA. Severity of small-for-gestational-age and morbidity and mortality among very preterm neonates. J Perinatol. (2023) 43(4):437-44. doi:10.1038/s41372-022-01544-w

171. Mohsin M, Wong F, Bauman A, Bai J. Maternal and neonatal factors influencing premature birth and low birth weight in Australia. Journal of Biosocial Science. (2003) 35(2):161-74. doi:10.1017/S0021932003001615

172. Mukhopadhyay K, Louis D, Mahajan R, Kumar P. Predictors of mortality and major morbidities in extremely low birth weight neonates. Indian Pediatr. (2013) 50(12):1119-23. doi:10.1007/s13312-013-0305-8

173. Ratiu D, Sauter F, Gilman E, Ludwig S, Ratiu J, Mallmann-Gottschalk N, et al. Impact of Advanced Maternal Age on Maternal and Neonatal Outcomes. In Vivo. (2023) 37(4):1694-702. doi:10.21873/invivo.13256

174. Ruth CA, Roos N, Hildes-Ripstein E, Brownell M. The influence of gestational age and socioeconomic status on neonatal outcomes in late preterm and early term gestation: A population based study. BMC Pregnancy and Childbirth. (2012) 12. doi:10.1186/1471-2393-12-62

175. Santomartino GA, Blank DA, Heng A, Woodward A, Kane SC, Thio M, et al. Perinatal predictors of clinical instability at birth in late-preterm and term infants. European Journal of Pediatrics. (2023) 182(3):987-95. doi:10.1007/s00431-022-04684-5

176. Shapiro-Mendoza CK, Tomashek KM, Kotelchuck M, Barfield W, Weiss J, Evans S. Risk Factors for Neonatal Morbidity and Mortality Among "Healthy," Late Preterm Newborns. Seminars in Perinatology. (2006) 30(2):54-60. doi:10.1053/j.semperi.2006.02.002

177. Sharma D, Murki S, Pratap T, Deshbotla SK, Vardhelli V, Pawale D, et al. Association between admission temperature and mortality and major morbidity in very low birth weight neonates–single center prospective observational study. Journal of Maternal-Fetal and Neonatal Medicine. (2022) 35(16):3096-104. doi:10.1080/14767058.2020.1810229

178. Shrestha S, Dangol Singh S, Shrestha M, Shrestha RP. Outcome of preterm babies and associated risk factors in a hospital. Journal of the Nepal Medical Association. (2010) 50(4):286-90. doi:10.31729/jnma.57

179. Sritipsukho S, Suarod T, Sritipsukho P. Survival and outcome of very low birth weight infants born in a university hospital with level II NICU. Journal of the Medical Association of Thailand = Chotmaihet thangphaet. (2007) 90(7):1323-9.

180. Stephens AS, Lain SJ, Roberts CL, Bowen JR, Simpson JM, Nassar N. Hospitalisations from 1 to 6 years of age: effects of gestational age and severe neonatal morbidity. Paediatric and perinatal epidemiology. (2015) 29(3):241-9. doi:10.1111/ppe.12188

181. Sun J, Qu S, Zhang C, Xiang Z, Fu Z, Yao L. Neonatal mortality rate and risk factors in northeast China: Analysis of 5,277 neonates in 2005. Clinical and Experimental Obstetrics and Gynecology. (2014) 41(5):512-6. doi:10.12891/ceog16662014

182. Tarnow-Mordi WO, Abdel-Latif ME, Martin A, Pammi M, Robledo K, Manzoni P, et al. The effect of lactoferrin supplementation on death or major morbidity in very low birthweight infants (LIFT): a multicentre, double-blind, randomised controlled trial. The lancet. Child & adolescent health. (2020) 4(6):444‐54. doi:10.1016/S2352-4642(20)30093-6

183. Tedesco RP, Passini Jr R, Cecatti JG, Camargo RS, Pacagnella RC, Sousa MH. Estimation of preterm birth rate, associated factors and maternal morbidity from a demographic and health survey in Brazil. Maternal and Child Health Journal. (2013) 17(9):1638-47. doi:10.1007/s10995-012-1177-6

184. Tembo T, Koyuncu A, Zhuo H, Mwendafilumba M, Manasyan A. The association of maternal age with adverse neonatal outcomes in Lusaka, Zambia: a prospective cohort study. BMC Pregnancy Childbirth. (2020) 20(1). doi:10.1186/s12884-020-03361-5

185. Walter-Nicolet E, Courtois E, Milesi C, Ancel PY, Beuchée A, Tourneux P, et al. Premedication practices for delivery room intubations in premature infants in France: Results from the EPIPAGE 2 cohort study. PLoS One. (2019) 14(4):e0215150. doi:10.1371/journal.pone.0215150

186. Wang Y, Tanbo T, Ellingsen L, Abyholm T, Henriksen T. Effect of pregestational maternal, obstetric and perinatal factors on neonatal outcome in extreme prematurity. Arch. Gynecol. Obstet. (2011) 284(6):1381-7. doi:10.1007/s00404-011-1870-5

187. Wilder R, Robinson C, Jared HL, Lieff S, Boggess K. Obstetricians' knowledge and practice behaviors concerning periodontal health and preterm delivery and low birth weight. J Dent Hyg. 2007;81(4):81.

188. Yorifuji T, Naruse H, Kashima S, Takao S, Murakoshi T, Doi H, et al. Residential proximity to major roads and adverse birth outcomes: a hospital-based study. Environ Health. (2013) 12. doi:10.1186/1476-069X-12-34

189. Zeitlin J, Egorova NN, Janevic T, Hebert PL, Lebreton E, Balbierz A, et al. The Impact of Severe Maternal Morbidity on Very Preterm Infant Outcomes. Journal of Pediatrics. (2019) 215:56-63.e1. doi:10.1016/j.jpeds.2019.07.061

190. Anand AJ, Sabapathy K, Sriram B, Rajadurai VS, Agarwal PK. Single Center Outcome of Multiple Births in the Premature and Very Low Birth Weight Cohort in Singapore. Am J Perinatol. 2022;39(4):409-415. doi:10.1055/s-0040-1716482

191. Blennow M, Ewald U, Fritz T, Holmgren PA, Jeppsson A, Lindberg E, et al. One-Year Survival of Extremely Preterm Infants After Active Perinatal Care in Sweden. JAMA. (2009) 301(21):2225-33. doi:10.1001/jama.2009.771

192. de Waal CG, Weisglas-Kuperus N, van Goudoever JB, Walther FJ, Neoned Study G, Grp LNFS. Mortality, Neonatal Morbidity and Two Year Follow-Up of Extremely Preterm Infants Born in the Netherlands in 2007. PLOS ONE. (2012) 7(7). doi:10.1371/journal.pone.0041302

193. Fanaroff AA, Stoll BJ, Wright LL, Carlo WA, Ehrenkranz RA, Stark AR, et al. Trends in neonatal morbidity and mortality for very low birthweight infants. Am J Obstet Gynecol. (2007) 196(2):8.

194. Kumar P, Shankaran S, Ambalavanan N, Kendrick DE, Pappas A, Vohr BR, et al. Characteristics of extremely low-birth-weight infant survivors with unimpaired outcomes at 30 months of age. J Perinatol. (2013) 33(10):800-5. doi:10.1038/jp.2013.71

195. Lee SK, Beltempo M, McMillan DD, Seshia M, Singhal N, Dow K, et al. Outcomes and care practices for preterm infants born at less than 33 weeks’ gestation: A quality-improvement study. CMAJ. (2020) 192(4):E81-E91. doi:10.1503/cmaj.190940

196. Norman M, Hallberg B, Abrahamsson T, Björklund LJ, Domellöf M, Farooqi A, et al. Association between Year of Birth and 1-Year Survival among Extremely Preterm Infants in Sweden during 2004-2007 and 2014-2016. JAMA. (2019) 321(12):1188-99. doi:10.1001/jama.2019.2021

197. Stensvold HJ, Klingenberg C, Stoen R, Moster D, Braekke K, Guthe HJ, et al. Neonatal morbidity and 1-year survival of extremely preterm infants. Pediatrics. (2017) 139(3). doi:10.1542/peds.2016-1821

198. Torchin H, Ancel PY, Jarreau PH, Goffinet F. Epidemiology of preterm birth: Prevalence, recent trends, short- and long-term outcomes. J Gynecol Obstet Biol Reprod (Paris). (2015) 44(8):723-31. doi:10.1016/j.jgyn.2015.06.010

199. Zhang T, Chen J, Wu H, Pan W, Yang X, Li Y, et al. Improved survival and survival without bronchopulmonary dysplasia in very low birth weight infants after active perinatal care. Niger J Clin Pract. (2020) 23(7):980-7. doi:10.4103/njcp.njcp_533_19

200. Kong X, Zhang S, Feng Z, Xu F, Tong X, Liu S, et al. Multicenter survey on early prognosis of extremely/very preterm infants in 14 hospitals in China. Chin. Med. J. (2018) 53(12):1356-62.

201. Xu F, Kong X, Duan S, Lv H, Ju R, Li Z, et al. Care Practices, Morbidity and Mortality of Preterm Neonates in China, 2013-2014: a Retrospective study. Sci. Rep. (2019) 9(1):19863. doi:10.1038/s41598-019-56101-x

202. Qiu LY, Yan JY, Liu H, Ren KH. Research on the Incidence Rate of Extremely Preterm Birth in China and Relationship between Extremely Preterm Birth and Chorioamnionitis. Indian J Pharm Sc. (2021) 83:42-8. doi:10.36468/pharmaceutical-sciences.spl.249
